# Risk Model–Based Lung Cancer Screening and Racial and Ethnic Disparities in the US

**DOI:** 10.1001/jamaoncol.2023.4447

**Published:** 2023-10-26

**Authors:** Eunji Choi, Victoria Y. Ding, Sophia J. Luo, Kevin ten Haaf, Julie T. Wu, Jacqueline V. Aredo, Lynne R. Wilkens, Neal D. Freedman, Leah M. Backhus, Ann N. Leung, Rafael Meza, Natalie S. Lui, Christopher A. Haiman, Sung-Shim Lani Park, Loïc Le Marchand, Joel W. Neal, Iona Cheng, Heather A. Wakelee, Martin C. Tammemägi, Summer S. Han

**Affiliations:** 1 Quantitative Sciences Unit, Stanford University School of Medicine, Stanford, California; 2 Department of Neurosurgery, Stanford University School of Medicine, Stanford, California; 3 Department of Public Health, Erasmus Medical Center, Rotterdam, the Netherlands; 4 Stanford University School of Medicine, Stanford, California; 5 Department of Medicine, University of California, San Francisco; 6 Cancer Epidemiology Program, University of Hawaii Cancer Center, Honolulu, Hawaii; 7 Division of Cancer Epidemiology and Genetics, National Cancer Institute, National Institutes of Health, Bethesda, Maryland; 8 Department of Cardiothoracic Surgery, Stanford University School of Medicine, Stanford, California; 9 Department of Radiology, Stanford University School of Medicine, Stanford, California; 10 Department of Epidemiology, School of Public Health, University of Michigan, Ann Arbor; 11 Center for Genetic Epidemiology, Department of Population and Public Health Sciences, Keck School of Medicine, University of Southern California, Los Angeles; 12 Division of Oncology, Department of Medicine, Stanford University School of Medicine, Stanford, California; 13 Stanford Cancer Institute, Stanford University School of Medicine, Stanford, California; 14 Department of Epidemiology and Biostatistics, University of California, San Francisco; 15 Department of Health Sciences, Brock University, St Catharines, Ontario, Canada; 16 Department of Epidemiology and Population Health, Stanford University School of Medicine, Stanford, California

## Abstract

**Importance:**

The revised 2021 US Preventive Services Task Force (USPSTF) guidelines for lung cancer screening have been shown to reduce disparities in screening eligibility and performance between African American and White individuals vs the 2013 guidelines. However, potential disparities across other racial and ethnic groups in the US remain unknown. Risk model–based screening may reduce racial and ethnic disparities and improve screening performance, but neither validation of key risk prediction models nor their screening performance has been examined by race and ethnicity.

**Objective:**

To validate and recalibrate the Prostate, Lung, Colorectal, and Ovarian Cancer Screening Trial 2012 (PLCOm2012) model—a well-established risk prediction model based on a predominantly White population—across races and ethnicities in the US and evaluate racial and ethnic disparities and screening performance through risk-based screening using PLCOm2012 vs the USPSTF 2021 criteria.

**Design, Setting, and Participants:**

In a population-based cohort design, the Multiethnic Cohort Study enrolled participants in 1993-1996, followed up through December 31, 2018. Data analysis was conducted from April 1, 2022, to May 19. 2023. A total of 105 261 adults with a smoking history were included.

**Exposures:**

The 6-year lung cancer risk was calculated through recalibrated PLCOm2012 (ie, PLCOm2012-Update) and screening eligibility based on a 6-year risk threshold greater than or equal to 1.3%, yielding similar eligibility as the USPSTF 2021 guidelines.

**Outcomes:**

Predictive accuracy, screening eligibility-incidence (E-I) ratio (ie, ratio of the number of eligible to incident cases), and screening performance (sensitivity, specificity, and number needed to screen to detect 1 lung cancer).

**Results:**

Of 105 261 participants (60 011 [57.0%] men; mean [SD] age, 59.8 [8.7] years), consisting of 19 258 (18.3%) African American, 27 227 (25.9%) Japanese American, 21 383 (20.3%) Latino, 8368 (7.9%) Native Hawaiian/Other Pacific Islander, and 29 025 (27.6%) White individuals, 1464 (1.4%) developed lung cancer within 6 years from enrollment. The PLCOm2012-Update showed good predictive accuracy across races and ethnicities (area under the curve, 0.72-0.82). The USPSTF 2021 criteria yielded a large disparity among African American individuals, whose E-I ratio was 53% lower vs White individuals (E-I ratio: 9.5 vs 20.3; *P* < .001). Under the risk-based screening (PLCOm2012-Update 6-year risk ≥1.3%), the disparity between African American and White individuals was substantially reduced (E-I ratio: 15.9 vs 18.4; *P* < .001), with minimal disparities observed in persons of other minoritized groups, including Japanese American, Latino, and Native Hawaiian/Other Pacific Islander. Risk-based screening yielded superior overall and race and ethnicity–specific performance to the USPSTF 2021 criteria, with higher overall sensitivity (67.2% vs 57.7%) and lower number needed to screen (26 vs 30) at similar specificity (76.6%).

**Conclusions:**

The findings of this cohort study suggest that risk-based lung cancer screening can reduce racial and ethnic disparities and improve screening performance across races and ethnicities vs the USPSTF 2021 criteria.

## Key Points

**Question:**

Can risk-based lung cancer screening reduce racial and ethnic disparities and improve screening efficiency vs the national lung cancer screening guidelines across 5 races and ethnicities in the US?

**Findings:**

In this cohort study including 105 261 adults with a smoking history, the 2021 US Preventive Services Task Force guidelines yielded a large disparity in African American individuals whose eligibility-incidence ratio was 53% lower than that of White individuals. Under risk-based screening, the disparity between African American and White individuals was reduced—with minimal disparities observed across other minoritized groups—and improved screening efficiency across races and ethnicities.

**Meaning:**

The findings of this study suggest that risk-based screening reduces racial and ethnic disparities while maintaining improved screening efficiency.

## Teaser

This cohort study evaluates use of risk-based screening for lung cancer with the Prostate, Lung, Colorectal, and Ovarian Cancer Screening Trial 2012 prediction model to address racial and ethnic disparities in individuals with a smoking history.

## Introduction

Lung cancer is the leading cause of cancer death in the US.^1^ The landmark lung cancer screening trials demonstrated more than 20% lung cancer–specific mortality reduction through low-dose computed tomography.^2,3^ Based on the trial evidence^2,3^ combined with modeling efforts,^4^ the US Preventive Services Task Force (USPSTF) issued guidelines for lung cancer screening in 2013.^5^ In 2021, the USPSTF updated these recommendations by lowering the starting age from 55 to 50 years and the minimum cumulative smoking exposure from 30 to 20 pack-years compared with the 2013 recommendation.^6^ Several studies reported that the revised criteria would reduce racial disparities between African American and White individuals regarding screening eligibility^7^ and screening performance^8^ compared with the 2013 USPSTF guidelines. However, the disparities among other minoritized groups in the US, such as Asian or Latino, have been poorly examined. While there are a few existing studies that included other ethnic minority groups, most were limited to case-only,^9,10^ cross-sectional,^11,12,13^ or retrospective study designs,^8^ thus lacking comprehensive analyses that are needed to evaluate racial and ethnic disparities under various eligibility criteria.

Risk model–based screening may improve screening performance^14,15,16,17,18^ and further reduce racial and ethnic disparities^19,20,21^ compared with strategies based only on age and smoking history (eg, the USPSTF recommendation). The Prostate, Lung, Colorectal, and Ovarian Cancer Screening Trial 2012 (PLCOm2012) model ^17,22,23,24^ is a validated risk prediction model for lung cancer and has been implemented in several screening programs worldwide.^22,25,26^ The performance of risk-based screening through PLCOm2012 has been documented with improved screening efficiency^14,15,16^ and the potential for reducing racial disparities between African American and White individuals.^19,20,21^ However, studies evaluating disparities and screening performance are lacking among other minority groups in the US. Moreover, racial and ethnic disparities have been mostly examined by evaluating differences in screening eligibility.^7,10,11,12^ However, eligibility itself in a certain racial or ethnic group might be an incomplete disparity indicator unless the actual cancer risk is taken into account. Thus, an ideal metric to assess lung cancer screening disparities by race and ethnicity needs to capture differences in both exposure (eligibility) and risk (lung cancer incidence).^21^

Several other risk prediction models for lung cancer also have shown good performance in terms of easy applicability and external validation, including the Lung Cancer-Risk Assessment Tool (LCRAT),^27^ the model by Bach,^28,29^ and the Liverpool Lung Project-Risk Stratification Model (LLPv3).^30^ However, as with PLCOm2012, these models were developed and validated in predominantly White populations, thereby raising a question on their predictive accuracy in other racial and ethnic groups.

In this cohort study using a prospective multiethnic population-based cohort, we examine the predictive performance of PLCOm2012 and evaluate racial and ethnic disparities and screening performance through risk-based screening using PLCOm2012 vs the revised USPSTF 2021 criteria across 5 racial and ethnic groups in the US. To better capture disparities by incorporating screening eligibility and lung cancer risk, we estimate the eligibility-incidence (E-I) ratio in each racial and ethnic group. Furthermore, we investigate the predictive performance of other leading risk prediction models by race to evaluate their applicability across different ethnicities.

## Methods

### Study Population: The Multiethnic Cohort Study

The Multiethnic Cohort Study (MEC) is a prospective cohort study of adults aged 45 to 75 years enrolled from the populations of California and Hawaii in 1993-1996 (eMethods 1 in Supplement 1). The participants are representative of 5 racial and ethnic groups (African American, Japanese American, Latino, Native Hawaiian/Other Pacific Islander, and White) in the US.^31^ The present study included 105 261 participants after excluding those without a smoking history and those with missing smoking or racial and ethnic data (eMethods 1 in Supplement 1). Exposure data, including smoking history, sociodemographic factors, and clinical characteristics, were collected from a self-reported questionnaire at cohort enrollment (1993-1996). Receipt of a questionnaire was recognized as consent to participate in the MEC by the institutional review boards of the University of Hawaii and the University of Southern California; all participating sites received a waiver of consent per institutional review board policy. Incident cancers were identified through linkage to the Surveillance, Epidemiology, and End Results program cancer registries through December 31, 2018 (eMethods 1 in Supplement 1).^31^ This study followed the Strengthening the Reporting of Observational Studies in Epidemiology (STROBE) reporting guideline for cohort studies.

### Overview of Study Outcomes

The outcomes of this study included (1) predictive performance (discrimination, calibration, and predictive accuracy); (2) screening eligibility through the USPSTF 2021 criteria vs risk-based screening using PLCOm2012; (3) E-I ratio under each eligibility criterion, defined as the ratio between the total number of eligible participants vs incident lung cancer cases; and (4) screening performance (sensitivity, specificity, and number needed to screen [NNS] to detect 1 lung cancer) (eFigure 1 in Supplement 1).

### Statistical Analysis

#### Validation of Risk Prediction Models for Lung Cancer

Data analysis was conducted from April 1, 2022, to May 19, 2023. We used PLCOm2012^26^ as the main a priori comparator with the USPSTF 2021 criteria. We examined the following metrics to validate predictive performance per racial and ethnic group: discrimination (area under the curve [AUC]), calibration (calibration plot and slope), and predictive accuracy (Brier Score) (eMethods 2 in Supplement 1). Other leading risk prediction models, such as LCRAT,^27^ the Bach model,^28^ and LLPv3,^30^ were also evaluated for race and ethnicity–specific predictive accuracy (eMethods 3 in Supplement 1).

#### Estimating Screening Eligibility

The USPSTF 2021 criteria^6^ recommend annual computed tomography screening for individuals aged 50 to 80 years with at least a 20 pack-year smoking history and within 15 years after smoking cessation for former smokers. For each individual in the MEC, we calculated eligibility through the USPSTF 2021 criteria using the information on age and smoking history collected at cohort enrollment. We assessed eligibility under the USPSTF 2013 guidelines as a sensitivity analysis, which recommended screening for persons aged 55 to 80 years with at least 30 smoking pack-years and within 15 years of smoking cessation.^5^

The PLCOm2012 model^17,24^ (hereafter, PLCOm2012-Original) predicts individuals’ risk of incident lung cancer within 6 years, developed using the data from 39 219 people with a smoking history in the PLCO Screening Trial’s control arm. This model includes 11 predictors, including race and ethnicity, smoking-related factors, and educational level (eMethods 4 in Supplement 1).^17,24^ Due to suboptimal model calibrations of PLCOm2012-Original observed for several racial and ethnic groups in the MEC, we reestimated the race and ethnicity–related parameters of PLCOm2012-Original using MEC data, keeping the rest of the model parameters unchanged (eMethods 5; eTable 1 in Supplement 1). We used this updated model (hereafter, PLCOm2012-Update) as the primary comparator with the USPSTF 2021 guidelines. As a sensitivity analysis, we used the PLCOm2012-Original and the 3-level race model (PLCOm2012-Race3L).^10^ The latter was recently proposed to overcome the lower sensitivity of PLCOm2012-Original in racial and ethnic minority subgroups; this model merges the Asian, Hispanic, and Native Hawaiian/Other Pacific Islander groups with the White group as a reference (eMethods 6; eTable 1 in Supplement 1).

We calculated the 6-year risk of incident lung cancer using PLCOm2012-Update for each participant in the MEC based on the data collected at cohort enrollment. Individuals were deemed eligible for lung cancer screening if their predicted 6-year risk equaled or exceeded 1.3%, a risk threshold selected to match the percentage of the population eligible according to the USPSTF 2021 criteria in the MEC. In addition, we imposed an age-related restriction so that individuals outside of the USPSTF 2021–defined age range (50-80 years at cohort enrollment) were deemed ineligible as in prior studies.^9,19^ In addition to the 1.3% risk threshold, we applied different risk thresholds (1%, 1.51%, 1.7%, and 2%) as sensitivity analyses (eMethods 7 in Supplement 1).^26,32,33^

#### Evaluating Racial and Ethnic Disparities in E-I Ratio

As the main metric to evaluate racial and ethnic disparities in lung cancer screening, we used the E-I ratio, a composite measure of eligibility and incidence.^21^ This metric captures how the eligibility mirrors the actual risk of lung cancer across different racial and ethnic groups to estimate the potential disparity.^21^ A lower E-I ratio in a given group could refer to underserved screening (eligibility) despite the high lung cancer risk (incidence), thus suggestive of disparity. We compared the E-I ratio between the White group and each of the 4 other groups and quantified the difference using the percent difference metric. Poisson regression was performed to compare the E-I ratios by race and ethnic groups under each eligibility criterion.^34,35,36^ The assumption of the Poisson regression was checked by evaluating the mean and the variance of the outcome of the model. We used the Bonferroni method to adjust for multiple testing for 4 comparisons across races and ethnicities, with a significance level of *P* = .0125 (*P* = .05/4) using paired, 2-sided testing.

#### Evaluating Screening Performance

We compared the screening performance of the USPSTF 2021 criteria vs the risk-based screening strategy (PLCOm2012-Update [6-year risk ≥1.3%]) in the overall cohort and by race and ethnicity using the following metrics: sensitivity (the number of screening-eligible participants among those who develop lung cancer), specificity (the number of screening-ineligible participants among non–lung cancer cases), and the NNS to detect 1 lung cancer. Data were analyzed using R, version 4.31 (R Foundation for Statistical Computing).

## Results

### Study Population

Of 105 261 participants (60 011 [57.0%] men; 45 250 [43.0%] women; mean [SD] age, 59.8 [8.7] years) with a smoking history, consisting of 19 258 (18.3%) African American, 27 227 (25.9%) Japanese American, 21 383 (20.3%) Latino, 8368 (7.9%) Native Hawaiian/Other Pacific Islander, and 29 025 (27.6%) White individuals, 1464 (1.4%) developed lung cancer within 6 years from enrollment. The 6-year lung cancer incidence was highest among African American individuals (2.2%), followed by White and Native Hawaiian/Other Pacific Islander (1.5%), Japanese American (1.2%), and Latino (0.7%) individuals (Table).

**Table.** Characteristics of Participants With a Smoking History at Cohort Enrollment (1993-1996) in the Multiethnic Cohort Study

Characteristic	Participants, No. (%)					
	Overall	African American	Japanese American	Latino	Native Hawaiian/Other Pacific Islander	White
Total, No. (row %)	105 261 (100)	19 258 (18.3)	27 227 (25.9)	21 383 (20.3)	8368 (7.9)	29 025 (27.6)
6-y Incident lung cancer	1464 (1.4)	432 (2.2)	315 (1.2)	159 (0.7)	125 (1.5)	433 (1.5)
Baseline characteristics						
Age, y						
Mean (SD)	59.8 (8.7)	61.0 (8.9)	60.6 (8.9)	59.9 (7.6)	56.2 (8.4)	59.1 (9.0)
Sex						
Male	60 011 (57.0)	8570 (44.5)	18 384 (67.5)	14 005 (65.5)	4124 (49.3)	14 928 (51.4)
Female	45 250 (43.0)	10 688 (55.5)	8843 (32.5)	7378 (34.5)	4244 (50.7)	14 097 (48.6)
Educational level						
Less than high school (11th grade or below)	17 849 (17.0)	2915 (15.1)	2369 (8.7)	9280 (43.4)	1215 (14.5)	2070 (7.1)
High school graduate (12th grade or above)	28 476 (27.1)	5360 (27.8)	8285 (30.4)	5147 (24.1)	3539 (42.3)	6145 (21.2)
Training after high school/vocational school	8211 (7.8)	1219 (6.3)	3604 (13.2)	1425 (6.7)	650 (7.8)	1313 (4.5)
Some college	24 968 (23.7)	5942 (30.9)	5182 (19.0)	3476 (16.3)	1811 (21.6)	8557 (29.5)
College graduate	13 866 (13.2)	1995 (10.4)	4848 (17.8)	1053 (4.9)	666 (8.0)	5304 (18.3)
Postgraduate/professional school	11 891 (11.3)	1827 (9.5)	2939 (10.8)	1002 (4.7)	487 (5.8)	5636 (19.4)
BMI						
Mean (SD)	26.8 (5.1)	28.2 (5.7)	24.9 (3.7)	27.8 (4.8)	29.1 (6.3)	26.2 (4.9)
History of cancer^a^	9179 (8.7)	1926 (10.0)	1928 (7.1)	1327 (6.2)	632 (7.6)	3366 (11.6)
Family history of lung cancer	6786 (6.4)	1218 (6.3)	1839 (6.8)	853 (4.0)	632 (7.6)	2244 (7.7)
Smoking status						
Former smoker	74 100 (70.4)	12 030 (62.5)	20 672 (75.9)	15 195 (71.1)	5192 (62.0)	21 011 (72.4)
Current smoker	31 161 (29.6)	7228 (37.5)	6555 (24.1)	6188 (28.9)	3176 (38.0)	8014 (27.6)
Smoking duration, y						
Mean (SD)	22.5 (12.5)	23.6 (12.3)	22.6 (12.2)	20.5 (13.0)	23.1 (12.0)	22.9 (12.6)
Smoking quit-years						
Mean (SD)	9.5 (8.6)	7.6 (8.2)	10.8 (8.5)	9.3 (8.5)	7.9 (8.4)	10.1 (8.7)
Smoking pack-years						
Mean (SD)	18.4 (15.9)	16.1 (13.7)	19.8 (15.7)	13.0 (13.6)	20.1 (15.9)	22.2 (17.7)
Smoking intensity (cigarettes/d)						
Mean (SD)	14.8 (8.3)	12.5 (7.1)	16.0 (8.0)	11.0 (7.3)	15.9 (8.2)	17.6 (8.7)
Eligibility for lung cancer screening						
USPSTF 2021	25 282 (24.0)	4115 (21.4)	6932 (25.5)	3360 (15.7)	2104 (25.1)	8771 (30.2)
USPSTF 2013	15 681 (14.9)	2486 (12.9)	4301 (15.8)	2110 (9.9)	1137 (13.6)	5647 (19.5)
PLCOm2012-Update (risk threshold ≥1.3%)^b^	25 284 (24.0)	6879 (35.7)	5837 (21.4)	2549 (11.9)	2071 (24.7)	7948 (27.4)

Abbreviations: BMI, body mass index (calculated as weight in kilograms divided by height in meters squared); PLCO, Prostate, Lung, Colorectal, and Ovarian Cancer Screening Trial; USPSTF, US Preventive Services Task Force.

^a^ Skin cancer was excluded.

^b^ Risk threshold was identified to match eligibility through the USPSTF 2021 (24.0%).

### Validation for Race-Specific Predictive Performance in the MEC

**Figure 1.**
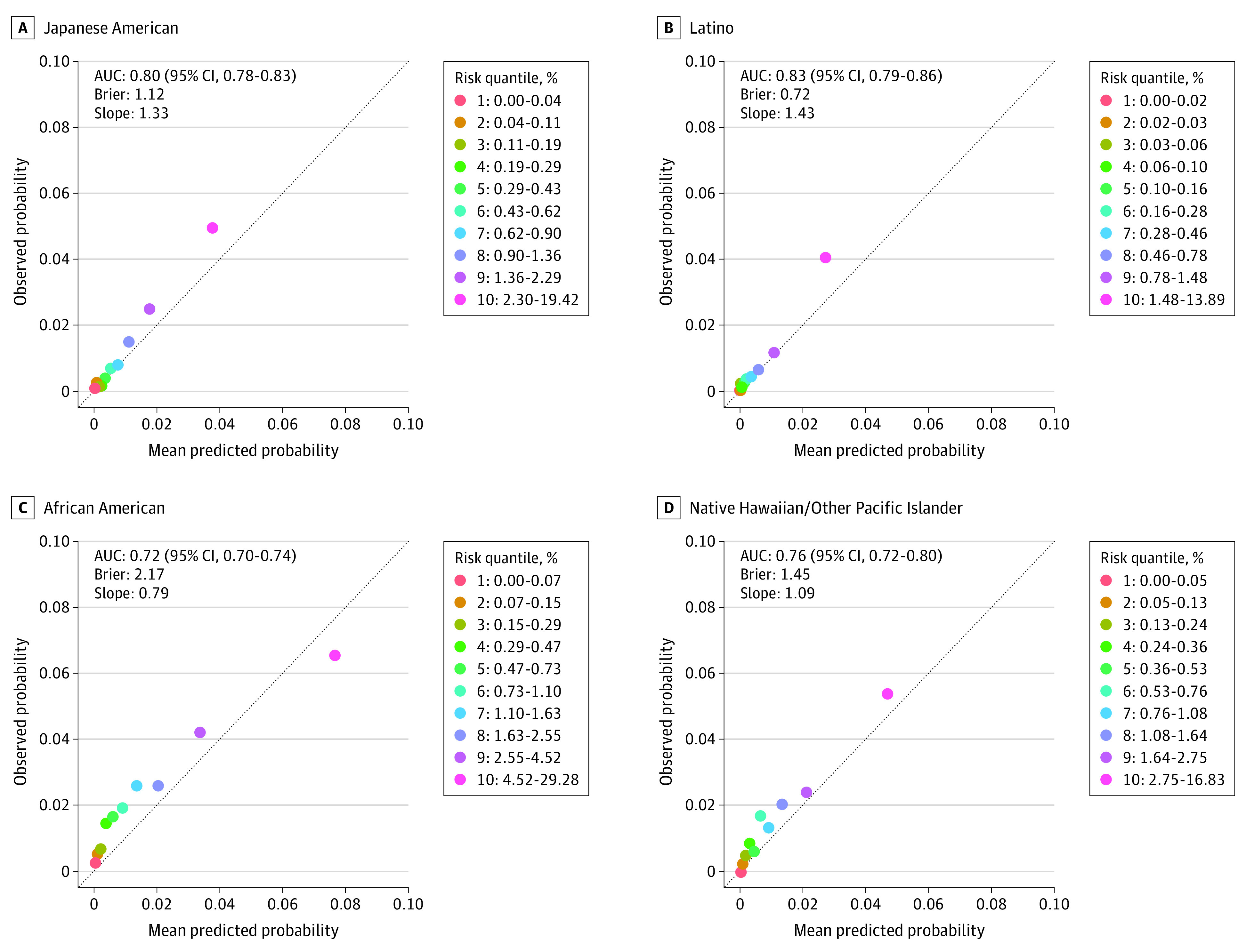
Predictive Performance of the Prostate, Lung, Colorectal, and Ovarian Cancer Screening Trial 2012 (PLCOm2012)-Update Model by Race and Ethnicity The discriminatory ability of the PLCOm2012-Update is evaluated by area under the curve (AUC). A, Japanese American (n = 27 727). B, Latino (n = 21 383). C, African American (n = 19 258). D, Native Hawaiian/Other Pacific Islander (n = 8368). Calibration between the observed and predicted probability of developing a 6-year lung cancer risk is presented with a calibration plot and calibration slope. The Brier score measures the accuracy of probabilistic predictions in the range of 0 and 1, and the lower the Brier score, the better the predictions are calibrated. All estimates are based on 10-fold cross-validation. Calibration measures the overall agreement between the observed and predicted outcomes by plotting a calibration curve between the observed vs predicted event status in groups by quantiles (eg, deciles) of the predicted probabilities. In this study, calibration ability was further quantified using the slope of the fitted linear regression between the means of observed and predicted probabilities across the decile groups. Perfect agreement between observed and predicted probability over deciles is shown by a slope of 1. The calibration slope is a simple, straightforward metric for evaluating overall calibration, but the graphic calibration plot across risk decile groups should also be taken into account because good calibration is dependent on a risk threshold of interest. The performance of PLCOm2012-Update on the White and overall cohorts is shown in eFigure 4 in Supplement 1.

The predictive performance of PLCOm2012-Original showed high overall discrimination, with an AUC of 0.79 (95% CI, 0.78-0.80). However, it posed a lack of calibration by underestimating risk among Japanese American (calibration slope: 1.66), Latino (calibration slope: 2.45), and Native Hawaiian/Other Pacific Islander (calibration slope: 1.39) participants (eFigure 2 in Supplement 1). The recalibrated model using MEC data (ie, PLCOm2012-Update) (eTable 1; eMethods 5 in Supplement 1) improved calibrations across all races and ethnicities (range of calibration slope: 0.79-1.43) (Figure 1; eFigure 3 in Supplement 1). Other risk prediction models (eFigures 4, 5, 6, and 7 in Supplement 1) showed clinically competent levels of AUC (>0.70) across all races and ethnicities, with LCRAT achieving the highest overall AUC of 0.80, yet with a lack of calibration among Native Hawaiian/Other Pacific Islander individuals (calibration slope: 1.95) (eFigure 5 in Supplement 1).

### Screening Eligibility and E-I Ratio

**Figure 2.**
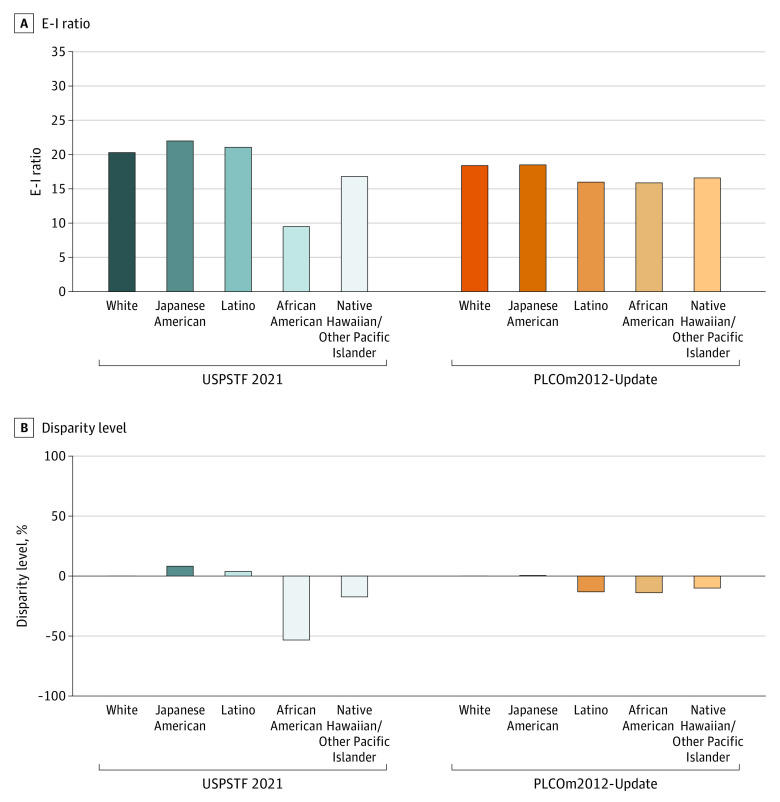
Racial Disparities in the Eligibility-Incidence (E-I) Ratio Through the US Preventive Services Task Force (USPSTF) 2021 and Risk-Based Screening (Prostate, Lung, Colorectal, and Ovarian Cancer Screening Trial 2012 [PLCOm2012]-Update Model 6-Year Risk ≥1.3%) Criteria A, Screening eligibility to 6-year lung cancer incidence (E-I) ratio is compared between the USPSTF 2021 criteria and risk-based criteria using the PLCOm2012-Update across different races and ethnicities. In risk-based screening, individuals were deemed eligible for lung cancer screening if their predicted 6-year risk of lung cancer using the PLCOm2012-Update equaled or exceeded the risk threshold (≥1.3%). B, The percent difference of the E-I ratio between the White and other racial and ethnic groups is calculated as follows: ([E-I ratio of a racial or ethnic group – E-I ratio of White] / [E-I ratio of White] x 100).

The overall eligibility for lung cancer screening through the USPSTF 2021 criteria was 24.0% among 105 261 MEC participants with a smoking history, with the highest eligibility observed in White individuals (30.2%), followed by Japanese American (25.5%), Native Hawaiian/Other Pacific Islander (25.1%), African American (21.4%), and Latino (15.7%) individuals (Table). The E-I ratio (ie, the ratio of the number of screening-eligible participants to incident cases) was 20.3 among White individuals under the USPSTF 2021 criteria, indicating that 20.3 screenings are provided under this criterion to detect 1 lung cancer case among White individuals. This E-I ratio was substantially lower among African American participants at 53% lower than among White participants (E-I ratio, 9.5 vs 20.3; *P* < .001) (Figure 2). The main source of this reduction in the E-I ratio among African American individuals was the lower eligibility (21.4% vs 30.2%) compared with White individuals under the USPSTF 2021 criteria yet a higher 6-year lung cancer incidence in African American (2.2%) compared with White (1.5%) individuals (Table; eTable 2 in Supplement 1). Among the other groups, a disparity between Native Hawaiian/Other Pacific Islander and White participants was observed, with a 17.2% lower E-I ratio among Native Hawaiian/Other Pacific Islander compared with White (16.8 vs 20.3; *P* < .001) participants under the USPSTF 2021 criteria.

Under the risk-based screening criteria using PLCOm2012-Update (6-year risk ≥1.3%), the difference in the E-I ratio between African American compared with White individuals was substantially reduced (E-I ratio: 15.9 vs 18.4; *P* < .001) relative to that with the USPSTF 2021 criteria (E-I ratio: 9.5 vs 20.3; *P* < .001) (Figure 2). This was mainly due to the increased eligibility among African American participants through the PLCOm2012-Update (35.7% vs 21.4% [USPSTF 2021]) (eTable 2 in Supplement 1). Furthermore, the disparity among Native Hawaiian/Other Pacific Islander compared with White individuals was reduced under risk-based screening (E-I ratio: 16.6 vs 18.4; *P* < .001) vs the USPSTF 2021 criteria (16.8 vs 20.3; *P* < .001) (Figure 2). Similar minimal differences in E-I ratio were observed in other ethnic groups compared with White individuals, including the Japanese American (E-I ratio: 18.5) group and Latino (E-I ratio: 16.0) group and compared with the White (E-I ratio: 18.4) group under risk-based screening (Figure 2).

### Screening Performance

**Figure 3.**
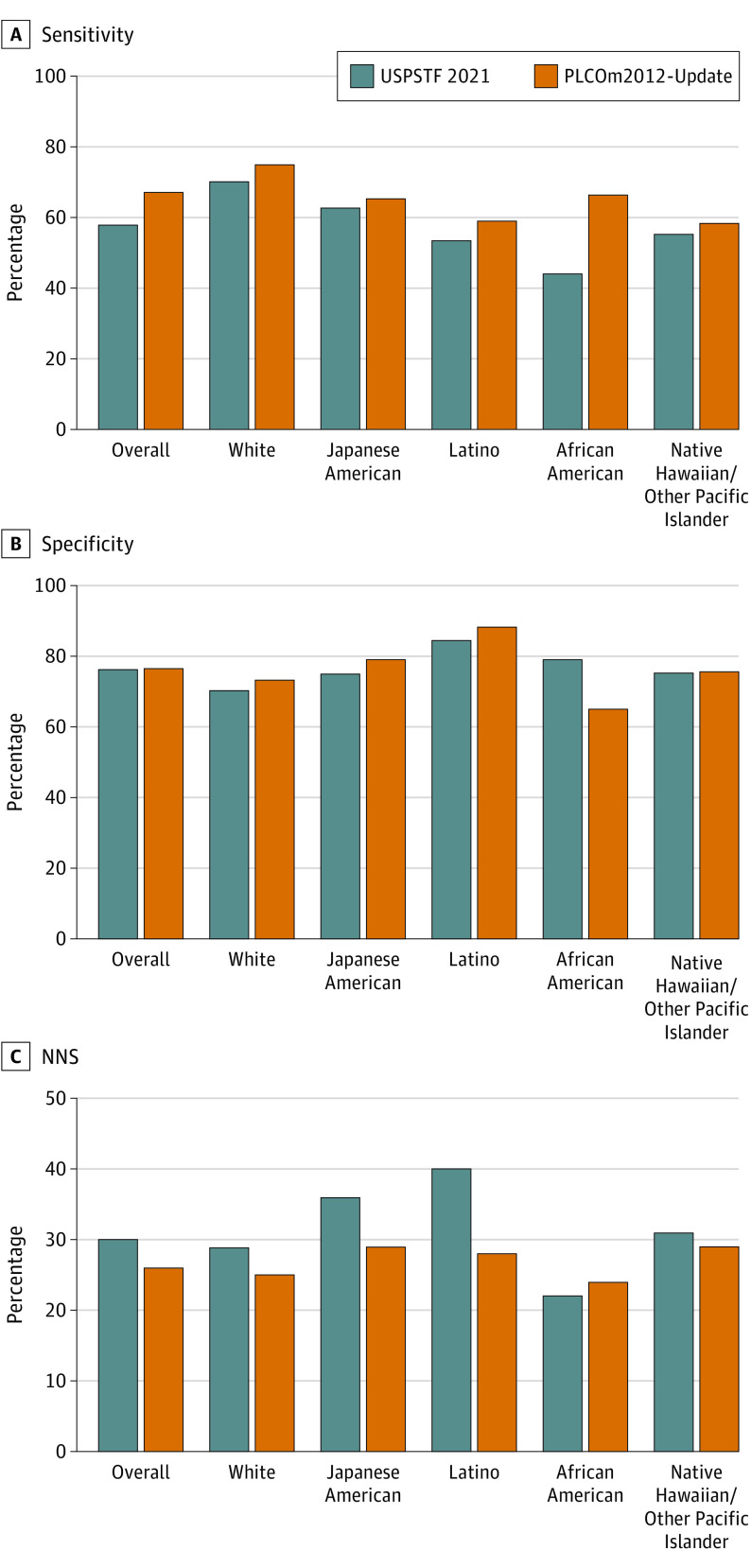
Screening Performance Through the US Preventive Services Task Force (USPSTF) 2021 and the Risk-Based Screening (Prostate, Lung, Colorectal, and Ovarian Cancer Screening Trial 2012 [PLCOm2012]-Update 6-Year Risk ≥1.3%) Criteria In the risk-based screening criteria, participants are eligible for screening if their predicted 6-year risk of lung cancer using the PLCOm2012-Update equals or exceeds the risk threshold (≥1.3%). A, Screening efficiency performance was quantified by screening sensitivity (the number of screening-eligible participants among lung cancer cases). B, Specificity (the number of screening-ineligible participants among non–lung cancer cases). C, The number needed to screen (NNS) to detect 1 lung cancer (the total number of screening-eligible cases divided by the number of screening-eligible lung cancer cases).

The risk-based screening through PLCOm2012-Update showed overall higher sensitivity (67.2% vs 57.7% [USPSTF 2021]) at a similar level of specificity of approximately 76% (Figure 3; eTable 3, eFigure 8 in Supplement 1). Similarly, risk-based screening showed higher screening efficiency with a smaller NNS (26 vs 30 [USPSTF 2021]) (Figure 3; eTable 3 in Supplement 1) in the overall cohort. Within each racial and ethnic group, sensitivity under the risk-based screening criteria was higher than the USPSTF 2021 criteria across all races and ethnicities (Figure 3). Specificity and NNS under risk-based screening were superior to the USPSTF 2021 criteria among all groups except for African American participants, among whom the USPSTF 2021 criteria showed slightly better specificity and NNS at the expense of decreased sensitivity and increased disparities in the E-I ratio vs risk-based screening.

### Sensitivity Analyses

The results under the alternative PLCOm2012 models (PLCOm2012-Original and PLCOm2012-Race3L) (eMethods 6 in Supplement 1) are shown in eTable 4 and eFigure 9 and eFigure 10 in Supplement 1. These alternative PLCOm2012 models produced a large eligibility gap, especially among the Latino and Native Hawaiian/Other Pacific Islander groups compared with the White group (eTable 4 in Supplement 1), thus showing exacerbated racial and ethnic disparities and reduced screening efficiency among Latino (in PLCOm2012-Original) and Native Hawaiian/Other Pacific Islander (in both the PLCOm2012-Original and PLCOm2012-Race3L models) individuals compared with the USPSTF 2021 criteria (eFigures 9 and 10 in Supplement 1). Sensitivity analyses under different risk thresholds yielded overall consistent findings as the primary analysis (eTable 5 in Supplement 1).

## Discussion

Based on data from a large, prospective multiracial and multiethnic cohort study, use of the USPSTF 2021 guidelines still resulted in racial and ethnic disparities in lung cancer screening. In particular, the E-I ratio that quantifies the screening-eligible vs incident cases was 53% lower among African American compared with White participants under the 2021 USPSTF guidelines—despite the increased eligibility among African American individuals (21.4% vs 12.9%)—compared with the 2013 USPSTF guidelines. This finding suggests that, while the revised USPSTF 2021 criteria doubled the eligible pool among African American individuals compared with the 2013 criteria, it does not fully cover the population at high risk of lung cancer. The disparity between these 2 groups was substantially reduced under the risk-based screening criteria through the PLCOm2012-Update (6-year risk ≥1.3%), with higher eligibility among African American individuals compared with the USPSTF 2021 criteria (PLCOm2012-Update, 35.7% vs USPSTF 2021, 21.4%). We also observed minimal disparities in E-I ratios under the risk-based screening strategy in other groups, including the Latino, Japanese American, and Native Hawaiian/Other Pacific Islander groups, compared with the White group. Risk-based screening also yielded superior overall screening performance to the USPSTF 2021 criteria, with higher overall sensitivity and lower NNS at similar specificity. Within each racial and ethnic group, sensitivity under the risk-based screening criteria was higher than in the USPSTF 2021 criteria across all groups.

The use of the E-I ratio—a composite metric that incorporates both eligibility and incidence vs eligibility alone—helped evaluate racial and ethnic disparities in lung cancer screening across groups of individuals who have potentially differential smoking exposures and baseline lung cancer risks. For example, recent studies reported markedly lower screening eligibility among Latino (16.2%-18.7%) compared with White (35.8%-40.9%) individuals under the USPSTF 2021 criteria, concluding that this finding represents a potential disparity.^12,13^ However, we found that the low screening eligibility among Latino compared with White individuals (15.7% vs 30.2%) under the USPSTF 2021 criteria reflects the low cumulative smoking exposure in the Latino population (mean pack-years, 13.0 vs 22.2) and the lowest 6-year incidence of lung cancer in Latino individuals (0.7% vs 1.5%) of all racial and ethnic groups in the MEC. When the E-I ratio was used, the difference between Latino compared with White participants was minimal (21.0 vs 20.3) in the MEC under the USPSTF 2021 criteria. Thus, this finding suggests that eligibility in a given racial or ethnic group is an incomplete disparity indicator unless the actual cancer risk is taken into account.

A recent study by Pinsky et al^21^ reported a relatively large disparity in the E-I ratio among Asian compared with White individuals under the USPSTF 2021 criteria, which was minimal in our study. This may be related to the fact that Pinsky et al used mixed data sources that included populations both with and without smoking histories to estimate the E-I ratio due to the use of the Surveillance, Epidemiology, and End Results program cancer registry that does not provide smoking data. However, in calculating the E-I ratio, the incident cases do not necessarily occur among the eligible participants. Thus, Asian individuals—who have a higher incidence of lung cancer among those without a smoking history^37^ but are not eligible for screening through the current screening guidelines—inevitably lowered the E-I ratio in the Pinsky et al^21^ study. In contrast, using an integrated data source with smoking information and cancer incidence from the MEC, we estimated the E-I ratio in a population with a smoking history only, focusing on the disparity through the current screening criteria. Although the ethnic composition of the Asian population differs in the prior study^21^ vs ours, this should not account for the minimal disparity observed in the MEC because lung cancer risk is similar across different Asian ethnicities with a smoking history.^38,39^

To our knowledge, this is the first study to externally and prospectively examine the performance of leading risk prediction models for lung cancer, including PLCOm2012-Original,^17,24^ LCRAT,^27^ the model by Bach,^28^ and LLPv3^30^ across 5 racial and ethnic groups in the US. Given that these lung cancer risk models were developed and validated using predominantly White populations,^17,23,27,28,29,30^ there is a critical need to assess their validity by race and ethnicity.

### Strengths and Limitations

Strengths of this study include the use of large racially and ethnically diverse prospective cohort data with a long follow-up for comprehensive cancer surveillance that included both participants with and without lung cancer, which enabled the evaluation of comprehensive screening performance metrics by race and ethnicity, including sensitivity, specificity, and NNS, as well as the E-I ratio. This approach contrasts with the existing studies based on retrospective,^8^ cross-sectional,^11,12,13^ or case-only data^9,10^ that were limited to evaluating eligibility^11,12,13^ or sensitivity alone^9^ or limited to using disjointed population-based data sets for estimating eligibility and lung cancer incidence.^21^

Despite the above-described strengths, the present study has several limitations. The population samples in the MEC collected from California and Hawaii might not reflect the entire US population structure. Although our findings suggest that risk-based screening could provide efficiency in detecting lung cancer and reducing disparities vs the USPSTF 2021 criteria, it remains unknown whether the efficacy of screening (ie, reduction in lung cancer mortality) potentially varies by racial or ethnic group. Future directions include investigating optimal risk thresholds for reducing lung cancer mortality by race and ethnicity, which may require extending existing microsimulation models^14,32^ to incorporate full risk factors for lung cancer, including race and ethnicity. Further research is warranted to investigate clinician- and individual-level barriers to undergoing lung cancer screening among individuals in high-risk racial and ethnic minority groups.

## Conclusions

Based on racially and ethnically diverse population-based cohort data, the 2021 USPSTF guidelines for lung cancer screening still induce racial and ethnic disparities. Risk-based lung cancer screening using a validated risk prediction model may help reduce racial and ethnic disparities in lung cancer screening and improve screening efficiency across racial and ethnic groups in the US.

## Notes

Supplement 1. **eMethods 1.** The Study Population **eMethods 2.** Evaluation of Predictive Performance of Lung Cancer Risk Prediction Models **eMethods 3.** Alternative Lung Cancer Risk Prediction Models **eMethods 4.** The Original PLCOm2012 Model (ie, PLCOm2012 _Original_) **eMethods 5.** The Recalibrated PLCOm2012 Model (ie, PLCOm2012 _Update_) **eMethods 6.** The PLCOm2012 _Race3L_ Model **eMethods 7.** Racial Disparities and Screening Performance in Risk-Based Screening Through PLCOm2012 _Update_ Using Different Risk Thresholds
eReferences
**eTable 1.** Coefficient Estimates From the PLCOm2012 Models **eTable 2.** Number of Total Participants, Screening Eligible, and 6-Year Lung Cancer Incidence to Calculate Eligibility to Incidence (E-I Ratio) in the USPSTF 2021 and Risk-Based Screening Criteria Through the PLCOm2012 _Update_ (6-Year Risk ≥1.3%) **eTable 3.** Screening Performance Under the USPSTF 2021 Criteria and the Risk-Based Screening Through the PLCOm2012 _Update_ (6-Year Risk ≥1.3%) **eTable 4.** Eligibility for Lung Cancer Screening Through the PLCOm2012 _Original_ and PLCOm2012 _Race3L_ **eTable 5.** Sensitivity Analyses for Racial Disparities in the E-I Ratio and Screening Performance Through the USPSTF 2021 and the Risk-Based Screening Criteria Using PLCOm2012 _Update_ at Alternative Risk Thresholds **eFigure 1.** A Schematic Diagram of Study Design **eFigure 2.** Predictive Performance of the PLCOm2012 _Original_ by Race **eFigure 3.** Predictive Performance of the PLCOm2012 _Update_ by Race **eFigure 4.** Predictive Performance of the PLCOm2012 _Race3L_ by Race **eFigure 5.** Predictive Performance of LCRAT by Race **eFigure 6.** Predictive Performance of the Bach Model by Race **eFigure 7.** Predictive Performance of LLPv3 by Race **eFigure 8.** Screening Performance (Positive and Negative Predictive Value) of the USPSTF 2021 Criteria and risk-Based Screening Through the PLCOm2012 _Update_ by Race **eFigure 9.** Sensitivity Analysis for Evaluating Racial Disparities in the Eligibility to Incidence (E-I) Ratio Through the USPSTF 2021 and Risk-Based Screening (PLCOm2012 _Original_ and PLCOm2012 _Race3L_ [6-year risk**≥**1.3%]) **eFigure 10.** Sensitivity Analysis for Screening Performance Through the USPSTF 2021 and Risk-Based Screening (PLCOm2012 _Original_ and PLCOm2012 _Race3L_ [6-year risk**≥**1.4%])

Supplement 2.
Data Sharing Statement
