# Supplementary material for: Risk Model–Based Lung Cancer Screening and Racial and Ethnic Disparities in the US
Source: JAMA Oncol. 2023 Oct 26;9(12):1640–8. doi: 10.1001/jamaoncol.2023.4447 (PMC10603577; doi:10.1001/jamaoncol.2023.4447)
Supplement: Supplement 1. — eMethods 1. The Study Population eMethods 2. Evaluation of Predictive Performance of Lung Cancer Risk Prediction Models eMethods 3. Alternative Lung Cancer Risk Prediction Models eMethods 4. The Original PLCOm2012 Model (ie, PLCOm2012 Original) eMethods 5. The Recalibrated PLCOm2012 Model (ie, PLCOm2012 Update) eMethods 6. The PLCOm2012 Race3L Model eMethods 7. Racial Disparities and Screening Performance in Risk-Based Screening Through PLCOm2012 Update Using Different Risk Thresholds eReferences eTable 1. Coefficient Estimates From the PLCOm2012 Models eTable 2. Number of Total Participants, Screening Eligible, and 6-Year Lung Cancer Incidence to Calculate Eligibility to Incidence (E-I Ratio) in the USPSTF 2021 and Risk-Based Screening Criteria Through the PLCOm2012 Update (6-Year Risk ≥1.3%) eTable 3. Screening Performance Under the USPSTF 2021 Criteria and the Risk-Based Screening Through the PLCOm2012 Update (6-Year Risk ≥1.3%) eTable 4. Eligibility for Lung Cancer Screening Through the PLCOm2012 Original and PLCOm2012 Race3L eTable 5. Sensitivity Analyses for Racial Disparities in the E-I Ratio and Screening Performance Through the USPSTF 2021 and the Risk-Based Screening Criteria Using PLCOm2012 Update at Alternative Risk Thresholds eFigure 1. A Schematic Diagram of Study Design eFigure 2. Predictive Performance of the PLCOm2012 Original by Race eFigure 3. Predictive Performance of the PLCOm2012 Update by Race eFigure 4. Predictive Performance of the PLCOm2012 Race3L by Race eFigure 5. Predictive Performance of LCRAT by Race eFigure 6. Predictive Performance of the Bach Model by Race eFigure 7. Predictive Performance of LLPv3 by Race eFigure 8. Screening Performance (Positive and Negative Predictive Value) of the USPSTF 2021 Criteria and risk-Based Screening Through the PLCOm2012 Update by Race eFigure 9. Sensitivity Analysis for Evaluating Racial Disparities in the Eligibility to Incidence (E-I) Ratio Through the USPSTF 2021 and Risk-Based Screening (PLCOm201 [file jamaoncol-e234447-s001.pdf]

Supplementary Online Content

Choi E, Ding VY, Luo SJ, et al. Risk model–based lung cancer screening and racial and ethnic disparities in the US. *JAMA Oncol*. Published online October 26, 2023. doi:10.1001/jamaoncol.2023.4447

eMethods 1. The Study Population
eMethods 2. Evaluation of Predictive Performance of Lung Cancer Risk Prediction Models
eMethods 3. Alternative Lung Cancer Risk Prediction Models
eMethods 4. The Original PLCOm2012 Model (ie, PLCOm2012 $_{\text{Original}}$)
eMethods 5. The Recalibrated PLCOm2012 Model (ie, PLCOm2012 $_{\text{Update}}$)
eMethods 6. The PLCOm2012 $_{\text{Race3L}}$ Model
eMethods 7. Racial Disparities and Screening Performance in Risk-Based Screening Through PLCOm2012 $_{\text{Update}}$ Using Different Risk Thresholds
eReferences
eTable 1. Coefficient Estimates From the PLCOm2012 Models
eTable 2. Number of Total Participants, Screening Eligible, and 6-Year Lung Cancer Incidence to Calculate Eligibility to Incidence (E-I Ratio) in the USPSTF 2021 and Risk-Based Screening Criteria Through the PLCOm2012 $_{\text{Update}}$ (6-Year Risk ≥1.3%)
eTable 3. Screening Performance Under the USPSTF 2021 Criteria and the Risk-Based Screening Through the PLCOm2012 $_{\text{Update}}$ (6-Year Risk ≥1.3%)
eTable 4. Eligibility for Lung Cancer Screening Through the PLCOm2012 $_{\text{Original}}$ and PLCOm2012 $_{\text{Race3L}}$
eTable 5. Sensitivity Analyses for Racial Disparities in the E-I Ratio and Screening Performance Through the USPSTF 2021 and the Risk-Based Screening Criteria Using PLCOm2012 $_{\text{Update}}$ at Alternative Risk Thresholds
eFigure 1. A Schematic Diagram of Study Design
eFigure 2. Predictive Performance of the PLCOm2012 $_{\text{Original}}$ by Race
eFigure 3. Predictive Performance of the PLCOm2012 $_{\text{Update}}$ by Race
eFigure 4. Predictive Performance of the PLCOm2012 $_{\text{Race3L}}$ by Race
eFigure 5. Predictive Performance of LCRAT by Race
eFigure 6. Predictive Performance of the Bach Model by Race
eFigure 7. Predictive Performance of LLPv3 by Race
eFigure 8. Screening Performance (Positive and Negative Predictive Value) of the USPSTF 2021 Criteria and risk-Based Screening Through the PLCOm2012 $_{\text{Update}}$ by Race
eFigure 9. Sensitivity Analysis for Evaluating Racial Disparities in the Eligibility to Incidence (E-I) Ratio Through the USPSTF 2021 and Risk-Based Screening (PLCOm2012 $_{\text{Original}}$ and PLCOm2012 $_{\text{Race3L}}$ [6-year risk≥1.3%])
eFigure 10. Sensitivity Analysis for Screening Performance Through the USPSTF 2021 and Risk-Based Screening (PLCOm2012 $_{\text{Original}}$ and PLCOm2012 $_{\text{Race3L}}$ [6-year risk≥1.4%])

This supplementary material has been provided by the authors to give readers additional information about their work.

© 2023 Choi E et al. *JAMA Oncology*.

eMethods 1. The study population

The Multiethnic Cohort Study (MEC) is a prospective cohort study of adults aged 45-75 years from the populations of Southern California and Hawaii in 1993-1996, designed to identify key factors associated with cancer risk across different racial groups. Exposure data, including smoking history, sociodemographic factors, and clinical characteristics, were collected from a self-reported questionnaire at cohort enrollment (1993-1996). Incident cancers were prospectively identified through linkage to two state-wide cancer registries (the Hawaii Tumor Registry and the California State Cancer Registry) of the SEER through 2018. Given the high quality of the cancer registry maintained by the SEER, the identification of incident cancer cases is nearly complete for those who still live in the catchment areas. Out-migration to states other than Hawaii and California in the MEC remains limited at roughly 3% after 7 years of follow-up from the cohort enrollment. The cohort members who have left the state are still being followed up on via follow-up surveys as well as linkages to Medicare, the National Death Index, and the cancer registries in other states through the SEER programs.

Of 214,862 participants enrolled in MEC, we included those belonging to five racial and ethnic groups (Whites, Japanese Americans, Latinos, African American, and Native Hawaiian), excluding "other" races (n=12,170).[1] We excluded those with never-smoking (n=82,856) or missing-smoking status (n=8,385)) because lung cancer screening is recommended to those who ever smoked in the U.S.[2,3] In addition, the individuals with missing values in the risk factors used in the PLCOm2012 model (n=6,190) were excluded to conduct a complete-case analysis given their low missing rates (3.1%) and the use of PLCOm2012 model as the primary comparator of this study. Finally, 105,261 MEC participants were included in this study.

© 2023 Choi E et al. *JAMA Oncology*.

eMethods 2. Evaluation of predictive performance of lung cancer risk prediction models

The predictive accuracy of $PLCOm2012_{update}$ across different racial and ethnic groups was evaluated by using three indicators: discrimination (area under the receiver operating characteristics curve [AUC])[4], calibration (calibration plot and slope)[5,6], and predictive accuracy (Brier Score)[7]. The AUC refers to the discriminative ability to distinguish individuals with or without incident lung cancers. Calibration measures the overall agreement between the observed and predicted outcomes by plotting a calibration curve between the observed versus predicted event status in groups by quantiles (e.g., deciles) of the predicted probabilities. In our study, calibration ability was further quantified using the slope of the fitted linear regression between the means of observed and predicted probabilities across the decile groups. Perfect agreement between observed and predicted probability over deciles is shown by a slope of 1. The calibration slope is a simple, straightforward metric for evaluating overall calibration, but the graphical calibration plot across risk decile groups should also be taken into account because good calibration is dependent on a risk threshold of interest. Overall prediction accuracy was summarized in the Brier score that assessed the deviation of risk predictions estimated by models from the observed rates.

© 2023 Choi E et al. *JAMA Oncology*.

eMethods 3. Alternative lung cancer risk prediction models

1. Lung Cancer-Risk Assessment Tool (LCRAT)

The LCRAT[8] cause-specific hazard model for estimating 5-year cumulative lung cancer incidence was developed using data from ever-smokers aged 55-74 years from the PLCO Cancer Screening Trial (1993-2009) control group and validated in the chest radiography groups of the PLCO and the NSLT (2002-2009). Models consisting of all categorical and continuous variables with all possible combinations of simple transformations were fitted, and the one with the lowest Akaike information criterion was selected as final. Covariates included age, education (6 levels), sex, race (Non-Hispanic White, Non-Hispanic black, Hispanic, Other), smoking intensity (cigarettes/day), smoking duration, smoking quit-years, body mass index, family history of lung cancer, and self-reported emphysema. Given the LCRAT model can be applied to any timeframe, we chose 6-year timeframe for comparability to other models (i.e., PLCOm2012, and the model by Bach).

In the present study, we assumed no history of emphysema (or anything related to lung function, such as COPD, bronchitis, etc.) for everyone, because this information in the Multiethnic Cohort Study (MEC) was only available through Medicare for participants aged 65 years and older. Moreover, we confirmed that the prevalence of COPD before incident lung cancer diagnosis within 6 years of cohort enrollment was very low (~1%) in the subset of the MEC cohort for participants aged 65 years and older. Risk estimation was implemented using the ‘lcrisks’ R package.[9]

2. The model developed by Bach et al. (Bach)

The Bach lung cancer risk prediction model was developed using data on 18,172 subjects enrolled in the Carotene and Retinol Efficacy Trial (CARET)—a large, randomized trial of lung cancer prevention.[10,11] Predictors (age, sex, asbestos exposure history, and smoking history) were chosen based on two criteria--identifiable from a clinical history and established or strongly suspected risk factors for lung cancer. Continuous predictors (age, smoking duration and quit-years, and cigarettes per day) were modeled with restricted cubic splines to allow for nonlinear or nonmonotonic effects, with knots placed at empirical quartiles. Two 1-year Cox proportional hazards regression models—one for predicting probability of lung cancer diagnosis and another for predicting probability of death without lung cancer—were developed to recursively estimate the absolute risk of lung cancer over multiple years, with values of the predictors and the at-risk pool reduced in each subsequent cycle to simulate one of two scenarios: continued smoking or continued abstinence from smoking. The final model was then validated in ever-smokers in a study of lung cancer screening with computed tomography.

In the present study, we estimated 6-year lung cancer risk and assumed no history of asbestos exposure for everyone, as this information was not collected in the MEC Study.

3. Version 3 of Liverpool Lung Project-Risk Stratification Model (LLPv3)

The original Liverpool Lung Project (LLP) model[12] was developed in 2008, and a second version (LLPv2)[13] was developed from case-control data in Liverpool and further adapted to identify high-risk subjects for intervention in the UK Lung Screening Trial. The third version, the model considered in the present study (LLPv3),[14] was calibrated to national figures and exhibited improvement in absolute lung cancer risk prediction. Both LLPv2 and LLPv3 were validated using questionnaire data from 75,958 individuals in the UK Lung Screening Trial over 5 years of follow-up for lung cancer. Predictors included age, sex, smoking duration (never, 1-19 years, 20-39 years, 40-59 years, 60 years or more), personal history of cancer, family history of lung cancer (none, before age 60, or on or after age 60), history of pneumonia or other lung conditions, and asbestos exposure. The risk score is calculated using coefficient estimates from this multivariable logistic regression model and an age- and sex-specific factor derived from

lung cancer incidence rates in the Liverpool area, then applying a sex-specific adjustment factor.

Because early (before age 60) or late onset (on/after age 60) of family history of lung cancer was not captured in MEC data, we assumed late onset for anyone with family history of lung cancer, which is consistent with findings in current literature. We also assumed no history of lung conditions or asbestos exposure for everyone.

© 2023 Choi E et al. *JAMA Oncology*.

eMethods 4. The original PLCOm2012 model (i.e., $PLCOm2012_{original}$)

The PLCOm2012 model (hereafter $PLCOm2012_{original}$) was developed with data from 39,219 ever-smokers in the control group of the Prostate, Lung, Colorectal, and Ovarian (PLCO) Cancer Screening Trial, and validated from 40,447 and 53,202 participants with a smoking history from the PLCO intervention group and the National Lung Screening Trial (NLST), respectively.[15,16] The model uses 11 predictors (age, race, education, body mass index [BMI], history of chronic obstructive pulmonary disease [COPD], prior history of cancer, family history of lung cancer, smoking status, cigarettes per day, smoking duration, and smoking quit-years) to predict the risk of incident lung cancer within 6 years. The estimated parameters of the predictors in PLCOm2012 are shown in **eTable 1**.

When calculating individual's 6-year risk of developing lung cancer using $PLCOm2012_{original}$, we used the Multiethnic Cohort Study (MEC) data collected from self-reported baseline questionnaire at cohort enrollment, with COPD assumed to be "no" for all individuals[17], as a history of COPD was only documented for participants aged 65 years and older. By assuming none having COPD, the predicted lung cancer risk through $PLCOm2012_{original}$ would be conservatively estimated; thus, the screening efficiency performance of the risk-based model through PLCOm2012 models in our study was also evaluated in a conservative way and the actual impact would be higher.

© 2023 Choi E et al. *JAMA Oncology*.

eMethods 5. The recalibrated PLCOm2012 model (i.e., $PLCOm2012_{update}$)

To address the lack of calibrations observed among minority ethnic/racial subgroups such as Latinos, Japanese Americans, and Native Hawaiians in MEC (see **eFigure 2**), we re-estimated the race-related parameters (**eTable 1**) of $PLCOm2012_{original}$ using the MEC data. We observed significant underestimation of the 6-year risk among Latinos and Japanese Americans and overestimation of the risk among Native Hawaiian using $PLCOm2012_{original}$. In re-estimating the four race-related parameters of $PLCOm2012_{original}$, we used the following procedure:

1. Dataset used for the $PLCOm2012_{original}$ recalibration: Among 214,862 healthy participants in the Multiethnic Cohort Study (MEC), we included those belonging to five racial/ethnic groups (White, Japanese American, Latino, African American, and Native Hawaiian), excluding "other" races (n=12,170). We excluded those with never- or missing-smoking status (n=91,241) because lung cancer screening is recommended to those who ever smoked in the U.S. In addition, the individuals with missing values in the risk factors used in the PLCOm2012 model (n=6,190) were excluded to conduct a complete-case analysis given their low missing rates (3.1%). Finally, 105,261 MEC participants were used for recalibration of the $PLCOm2012_{original}$.

2. Construction of a linear predictor (LP) except race-related parameters: We calculated a linear predictor (LP) using the coefficients and model constant of the $PLCOm2012_{original}$ model (**eTable 1**) except the race-related parameters based on the MEC dataset. The formula is provided below:

$$\begin{aligned} LP = -4.532506 &+ 0.0778868 * (Age - 62) \\ &- 0.0812744 * (Education - 4) \\ &- 0.0274194 * (BMI - 27) \\ &+ 0.3553063 * COPD \\ &+ 0.4589971 * Personal\ history\ of\ cancer \\ &+ 0.587185 * Family\ history\ of\ lung\ cancer \\ &+ 0.2597431 * Smoking\ status \\ &- 1.822606 * ((Smoking\ intensity/10)^{(-1)} - 0.4021541613) \\ &+ 0.0317321 * (Smoking\ duration - 27) \\ &- 0.0308572 * (Smoking\ quit\ time - 10)) \end{aligned}$$

3. Refitted logistic regression model: We refitted logistic regression for predicting the 6-year risk of lung cancer (Event: 6-year incidence of lung cancer; Time: Time from cohort enrollment to 6-year incidence of lung cancer) using a race variable (five levels) and a linear predictor variable as follows:
   1) LP
   2) A race variable of five levels (White, Japanese American, Latino, African American, and Native Hawaiian) using 'White' as a reference

   The results for the parameter estimates are provided below, which is also summarized in **eTable 1** in comparison to $PLCOm2012_{original.}$

	Estiamte	Std. Error	z-value	Pr(>|z|)
(Constant)	-0.50799	0.10459	-4.857	1.19e-06 ***
LP	0.79887	0.02321	34.424	< 2e-16 ***
African American	0.61576	0.06972	8.832	< 2e-16 ***
Latino	-0.19241	0.09448	-2.037	0.04169 *
Japanese American	-0.23382	0.07516	-3.111	0.00186 **

© 2023 Choi E et al. *JAMA Oncology*.

Native Hawaiian	0.26207	0.10360	2.530	0.01142 *

4. Model internal validation & 6-year lung cancer estimation: The evaluation of $PLCOm2012_{update}$ was performed through an internal validation technique using a 10-fold cross-validation in MEC. When calculating individual's 6-year risk of developing lung cancer using $PLCOm2012_{update}$, we used the MEC data collected at cohort enrollment, with COPD assumed to be "no" for all individuals,[17] as a history of COPD was only documented in the MEC participants aged 65 years and older. By assuming none having COPD, the predicted lung cancer risk through $PLCOm2012_{update}$ would be conservatively estimated.

© 2023 Choi E et al. *JAMA Oncology*.

eMethods 6. The PLCOm2012$_{race3L}$ model

PLCOm2012$_{race3L}$ is a recently proposed model with reduced racial categories as another approach to overcome the lower sensitivity of PLCOm2012$_{original}$ in racial/ethnic minority subgroups (**eTable 1**).[18] The PLCOm2012$_{race3L}$ model[18] is equivalent to the PLCOm2012$_{original}$ model, except with racial groups reparametrized from six to three categories, i.e., Black, indigenous (American Indian/Alaskan Native), and a single category for White, Asian, Hispanic, and other race.

© 2023 Choi E et al. *JAMA Oncology*.

eMethods 7. Racial disparities and screening performance in risk-based screening through $PLCOm2012_{update}$ using different risk thresholds

To provide a fair comparison between the 2021 USPSTF criteria and risk-based screening using $PLCOm2012_{update}$, we chose the 1.3% risk threshold to match the percentage of the population eligible for the 2021 USPSTF criteria in our study cohort, the Multiethnic Cohort Study (MEC). We also employed a set of widely-used alternative risk thresholds (1%, 1.51%, 1.7%, and 2.0%) for sensitivity analyses (**eTable 5**). The use of these alternative risk thresholds in a sensitivity analysis yielded overall consistent results, showing reduced racial disparities and improved screening performance in risk-based screening ($PLCOm2012_{update}$) vs. the USPSTF 2021 criteria (**eTable 5**).

Racial disparities in lung cancer screening, which were measured by the E-I ratio between Whites and each of the other racial groups (Japanese Americans, African Americans, and Native Hawaiians), were smaller in risk-based screening using $PLCOm2012_{update}$ vs. the USPSTF 2021 criteria across all risk threshold levels (**eTable 5**). Latinos, however, showed increased disparities under the extreme risk thresholds (1.7% and 2.0%), which was expected given their low screening eligibility due to Latino's low cumulative smoking exposures. In terms of screening performance, the use of different risk thresholds showed a trade-off between sensitivity and specificity, as expected, but the number needed to screen (NNS) to detect one lung cancer was lower in risk-based screening than the USPSTF 2021 criteria across all risk thresholds (**eTable 5**).

© 2023 Choi E et al. *JAMA Oncology*.

eReferences

1. Kolonel LN, Henderson BE, Hankin JH, et al. A multiethnic cohort in Hawaii and Los Angeles: baseline characteristics. *Am J Epidemiol.* 2000;151(4):346-357.
2. Moyer VA. Screening for lung cancer: U.S. Preventive Services Task Force recommendation statement. *Ann Intern Med.* 2014;160(5):330-338.
3. Krist AH, Davidson KW, Mangione CM, et al. Screening for Lung Cancer: US Preventive Services Task Force Recommendation Statement. *Jama.* 2021;325(10):962-970.
4. Tripepi G, Jager KJ, Dekker FW, Zoccali C. Diagnostic methods 2: receiver operating characteristic (ROC) curves. *Kidney international.* 2009;76(3):252-256.
5. Shipe ME, Deppen SA, Farjah F, Grogan EL. Developing prediction models for clinical use using logistic regression: an overview. *Journal of thoracic disease.* 2019;11(Suppl 4):S574.
6. Miller ME, Hui SL, Tierney WM. Validation techniques for logistic regression models. *Statistics in medicine.* 1991;10(8):1213-1226.
7. Rufibach K. Use of Brier score to assess binary predictions. *Journal of clinical epidemiology.* 2010;63(8):938-939.
8. Katki HA, Kovalchik SA, Berg CD, Cheung LC, Chaturvedi AK. Development and Validation of Risk Models to Select Ever-Smokers for CT Lung Cancer Screening. *Jama.* 2016;315(21):2300-2311.
9. Cheung LC, Kovalchik SA, Katki HA, Cheung MLC. Package ‘lcrisks’. 2021.
10. Bach PB, Kattan MW, Thornquist MD, et al. Variations in lung cancer risk among smokers. *J Natl Cancer Inst.* 2003;95(6):470-478.
11. Cronin KA, Gail MH, Zou Z, Bach PB, Virtamo J, Albanes D. Validation of a model of lung cancer risk prediction among smokers. *J Natl Cancer Inst.* 2006;98(9):637-640.
12. Cassidy A, Myles JP, van Tongeren M, et al. The LLP risk model: an individual risk prediction model for lung cancer. *Br J Cancer.* 2008;98(2):270-276.
13. Field JK, Duffy SW, Baldwin DR, et al. UK Lung Cancer RCT Pilot Screening Trial: baseline findings from the screening arm provide evidence for the potential implementation of lung cancer screening. *Thorax.* 2016;71(2):161-170.
14. Field JK, Vulkan D, Davies MPA, Duffy SW, Gabe R. Liverpool Lung Project lung cancer risk stratification model: calibration and prospective validation. *Thorax.* 2021;76(2):161-168.
15. Tammemägi MC, Church TR, Hocking WG, et al. Evaluation of the lung cancer risks at which to screen ever- and never-smokers: screening rules applied to the PLCO and NLST cohorts. *PLoS Med.* 2014;11(12):e1001764.
16. Tammemägi MC, Katki HA, Hocking WG, et al. Selection criteria for lung-cancer screening. *N Engl J Med.* 2013;368(8):728-736.
17. Ten Haaf K, Bastani M, Cao P, et al. A Comparative Modeling Analysis of Risk-Based Lung Cancer Screening Strategies. *J Natl Cancer Inst.* 2020;112(5):466-479.
18. Pasquinelli MM, Tammemägi MC, Kovitz KL, et al. Addressing Sex Disparities in Lung Cancer Screening Eligibility: USPSTF vs PLCOm2012 Criteria. *Chest.* 2022;161(1):248-256.

© 2023 Choi E et al. *JAMA Oncology*.

eTable 1. Coefficient estimates from the PLCOm2012 models

Variables	$PLCOm2012_{original}$	$PLCOm2012_{update}$	$PLCOm2012_{race3L}$
Age[a] (years)	0.0778868	0.0778868	0.0778868
Race			
White	Referent group	Referent group	Referent group
Hispanic	-0.7434744	**-0.19241**[c]	Referent group
Asian	-0.466585	**-0.23382**[c]	Referent group
American Indian or Alaskan Native	1.027152	**0**[c]	1.055978
Black	0.3944778	**0.61576**[c]	0.427964
Native Hawaiian or Pacific Islander	0	**0.26207**[c]	Referent group
Education[a] (levels 1-6)	-0.0812744	-0.0812744	-0.0812744
Body mass index[a] (kg/m^2)	-0.0274194	-0.0274194	-0.0274194
Chronic obstructive pulmonary disease (1=yes; 0=no)	0.3553063	0.3553063	0.3553063
Personal history of cancer (1=yes; 0=no)	0.4589971	0.4589971	0.4589971
Family history of lung cancer (1=yes; 0=no)	0.587185	0.587185	0.587185
Smoking status (1=current; 0=former)	0.2597431	0.2597431	0.2597431
Smoking intensity[b] (average cigarettes/day)	-1.822606	-1.822606	-1.822606
Smoking duration[a] (years)	0.0317321	0.0317321	0.0317321
Smoking quit time[a] (years)	-0.0308572	-0.0308572	-0.0308572
Model constant	-4.532506	-4.532506	-4.532506

a Age is centered on 62 years, education is centered on level 4, body mass index is centered on 27, smoking duration was centered on 27 years, and smoking quit time was centered on 10 years.
b Smoking intensity (average number of cigarettes smoked per day) had a nonlinear association with lung cancer and was transformed as follows: first scaled by dividing by 10, then exponentiated by the power -1 and centered by subtracting the quantity by 0.4021541613.
c The coefficients updated in $PLCOm2012_{update}$ compared to the $PLCOm2012_{original}$

© 2023 Choi E et al. *JAMA Oncology*.

eTable 2. Number of total participants, screening eligible, and 6-year lung cancer incidence to calculate eligibility to incidence (E-I) ratio in the USPSTF 2021 and risk-based screening criteria through the $PLCOm2012_{update}$ (6-year risk ≥1.3%)

	Overall	White	Japanese American	Latino	African American	Native Hawaiian
**The USPSTF 2021 criteria**						
No. of total population	105,261	29,025	27,227	21,383	19,258	8,368
No. of eligible cases	25,282	8,771	6,932	3,360	4,115	2,104
No. of incident cases[a]	1464	433	315	159	432	125
Eligibility (%)	24.0	30.2	25.5	15.7	21.4	25.1
6-year Incidence rate (%)	1.4	1.5	1.2	0.7	2.2	1.5
E-I ratio[b]	17.3	20.3	22.0	21.1	9.5	16.8
**$PLCOm2012_{update}$ criteria (6-year risk ≥1.3%)**						
No. of total population	105,261	29,025	27,227	21,383	19,258	8,368
No. of eligible cases	25,283	7,948	5,837	2,549	6,879	2,071
No. of incident cases[a]	1,464	433	315	159	432	125
Eligibility (%)	24.0	27.4	21.4	11.9	35.7	24.7
6-year Incidence rate (%)	1.4	1.5	1.2	0.7	2.2	1.5
E-I ratio[b]	17.3	18.4	18.5	16.0	15.9	16.6

Abbreviations: EI-Ratio, eligibility-incidence ratio; PLCO, Prostate, Lung, Colorectal, and Ovarian Cancer Screening Trial; USPSTF, the United States Preventive Services Task Force

a Incident lung cancer cases developed for 6 years from the cohort enrollment

b Eligibility to incidence (E-I) ratio is calculated as the eligible cases defined by the lung cancer screening eligibility criteria, divided by 6-year lung cancer incident cases.

© 2023 Choi E et al. *JAMA Oncology*.

eTable 3. Screening performance under the USPSTF 2021 criteria and the risk-based screening through the PLCOm2012$_{update}$ (6-year risk ≥1.3%)

	Overall	White	Japanese American	Latino	African American	Native Hawaiian
**The USPSTF 2021 criteria**						
No. of total population	105,261	29,025	27,227	21,383	19,258	8,368
No. of eligible cases	25,282	8,771	6,932	3,360	4,115	2,104
(a) No. screen-eligible with incident LC[a]	845	304	197	85	190	69
(b) No. screen-eligible without incident LC	24437	8467	6735	3275	3925	2035
(c) No. screen-ineligible with incident LC[a]	619	129	118	74	242	56
(d) No. screen-ineligible without incident LC	79360	20125	20177	17949	14901	6208
Sensitivity (%) = (a)/[(a)+(c)]	57.7	70.2	62.5	53.5	44.0	55.2
Specificity (%) = (d)/[(b)+(d)]	76.5	70.4	75.0	84.6	79.2	75.3
PPV (%) = (a)/[(a)+(b)]	3.3	3.5	2.8	2.5	4.6	3.3
NPV (%) = (d)/[(c)+(d)]	99.2	99.4	99.4	99.6	98.4	99.1
NNS (n) = [(a)+(b)]/(a)	30	29	36	40	22	31
**PLCOm2012$_{update}$ criteria** (6-year risk≥1.3%)						
No. of total population	105,261	29,025	27,227	21,383	19,258	8,368
No. of eligible cases	25284	7948	5837	2549	6879	2071
(a) No. screen-eligible with incident LC	984	324	206	94	287	73
(b) No. screen-eligible without incident LC	24300	7624	5631	2455	6592	1998
(c) No. screen-ineligible with incident LC	480	109	109	65	145	52
(d) No. screen-ineligible without incident LC	79497	20968	21281	18769	12234	6245
Sensitivity (%) = (a)/[(a)+(c)]	67.2	74.8	65.4	59.1	66.4	58.4
Specificity (%) = (d)/[(b)+(d)]	76.6	73.3	79.1	88.4	65	75.8
PPV (%) = (a)/[(a)+(b)]	3.9	4.1	3.5	3.7	4.2	3.5
NPV (%) = (d)/[(c)+(d)]	99.4	99.5	99.5	99.7	98.8	99.2
NNS (n) = [(a)+(b)]/(a)	26	25	29	28	24	29

Abbreviations: LC, lung cancer; NNS, number needed to screen to detect one lung cancer; NPV, negative predictive value; PLCO, Prostate, Lung, Colorectal, and Ovarian Cancer Screening Trial; PPV, positive predictive value; USPSTF, the United States Preventive Services Task Force

a Incident lung cancer cases developed for 6 years from the cohort enrollment

© 2023 Choi E et al. *JAMA Oncology*.

eTable 4. Eligibility for lung cancer screening through the $PLCOm2012_{original}$ and $PLCOm2012_{race3L}$

	Overall	White	Japanese American	Latino	African American	Native Hawaiian
**Total, n (Row %)**	105261(100.0)	29025 (27.6)	27227 (25.9)	21383 (20.3)	19258 (18.3)	8368 (7.9)
**Eligibility for lung cancer screening, n (%)**						
$PLCOm2012_{original}$ (≥1.1%)[a]	25283 (24.0)	9305 (32.1)	5645 (20.7)	1606 (7.5)	6776 (35.2)	1951 (23.3)
$PLCOm2012_{race3L}$ (≥1.3%)[a]	25282 (24.0)	7726 (26.6)	7103 (26.1)	3042 (14.2)	5842 (30.3)	1569 (18.8)
**Other thresholds ($PLCOm2012_{original}$)**						
≥1% (USPSTF2021 from CISNET)	26315 (25.0)	9637 (33.2)	5939 (21.8)	1728 (8.1)	6969 (36.2)	2042 (24.4)
≥1.51% (Mortality reduction)	17743 (16.9)	6818 (23.5)	3662 (13.4)	836 (3.9)	5092 (26.4)	1335 (16.0)
≥1.7% (USPSTF2013 from CISNET)	15479 (14.7)	6015 (20.7)	3050 (11.2)	665 (3.1)	4597 (23.9)	1152 (13.8)
≥2% (Stringent threshold)	12738 (12.1)	5075 (17.5)	2345 (8.6)	456 (2.1)	3941 (20.5)	921 (11.0)
**Other lung cancer risk models**						
LCRAT (≧1.2%)[b]	25281 (24.0)	8528 (29.4)	5685 (20.9)	2942 (13.8)	6851 (35.6)	1275 (15.2)
Bach (≧1.2%)[b]	25266 (24.0)	8014 (27.6)	6839 (25.1)	3959 (18.5)	4713 (24.5)	1741 (20.8)
LLPv3 (≧0.9%)[b]	25391 (24.1)	7321 (25.2)	6859 (25.2)	4155 (19.4)	5638 (29.3)	1418 (16.9)

Abbreviations: CISNET, Cancer intervention and surveillance modeling network; PLCO, Prostate, Lung, Colorectal, and Ovarian Cancer Screening Trial; USPSTF, the United States Preventive Services Task Force

a Risk threshold identified to match the eligibility through the USPSTF 2021 (24.0%)

© 2023 Choi E et al. *JAMA Oncology*.

eTable 5. Sensitivity analyses for racial disparities in the E-I ratio and screening performance through the USPSTF 2021 and the risk-based screening criteria using PLCOm2012$_{update}$ at alternative risk thresholds

	Racial disparity		Screening performance		
	EI-Ratio	Disparity level	Sensitivity	Specificity	NNS
**The 2021 USPSTF criteria**					
Overall	-	-	57.7	76.5	30
White	20.3	0	70.2	70.4	29
Japanese American	22	8.4	62.5	75.0	36
Latino	21.1	3.9	53.5	84.6	40
African American	9.5	-53.2	44.0	79.2	22
Native Hawaiian	16.8	-17.2	55.2	75.3	31
**PLCOm2012update (≧1.0%)**					
Overall	-	-	73.4	71.4	29
White	22.3	0	81.1	67.5	28
Japanese American	23.5	5.4	74.0	73.4	32
Latino	21	-5.8	62.3	84.7	34
African American	18.3	-17.9	71.8	59.6	26
Native Hawaiian	20.1	-9.9	64.8	70.4	32
**PLCOm2012update (≧1.51%)**					
Overall	-	-	62.3	80.1	24
White	15.7	0	69.7	77.2	23
Japanese American	15.3	-2.5	61.6	82.8	25
Latino	13.1	-16.6	52.8	90.6	25
African American	14.1	-10.2	61.1	69.0	24
Native Hawaiian	14.2	-9.6	54.4	79.2	27
**PLCOm2012update (≧1.7%)**					
Overall	-	-	58.3	82.4	23
White	13.9	0	65.6	80.0	22
Japanese American	13.3	-4.3	57.1	85.0	24
Latino	11.1	-20.1	47.8	92.0	24
African American	12.8	-7.9	57.6	72.0	23
Native Hawaiian	12.6	-9.4	51.2	81.7	25
**PLCOm2012update (≧2.0%)**					
Overall	-	-	52.9	85.4	21
White	11.7	0	57.5	83.1	21
Japanese American	10.8	-7.7	50.8	87.9	22
Latino	8.6	-26.5	43.4	93.9	20
African American	11.1	-5.1	54.6	75.7	21
Native Hawaiian	10.4	-11.1	48.8	85.0	22

Abbreviations: PLCO, Prostate, Lung, Colorectal, and Ovarian Cancer Screening Trial; USPSTF, the United States Preventive Services Task Force

© 2023 Choi E et al. *JAMA Oncology*.

eFigure 1. A schematic diagram of study design

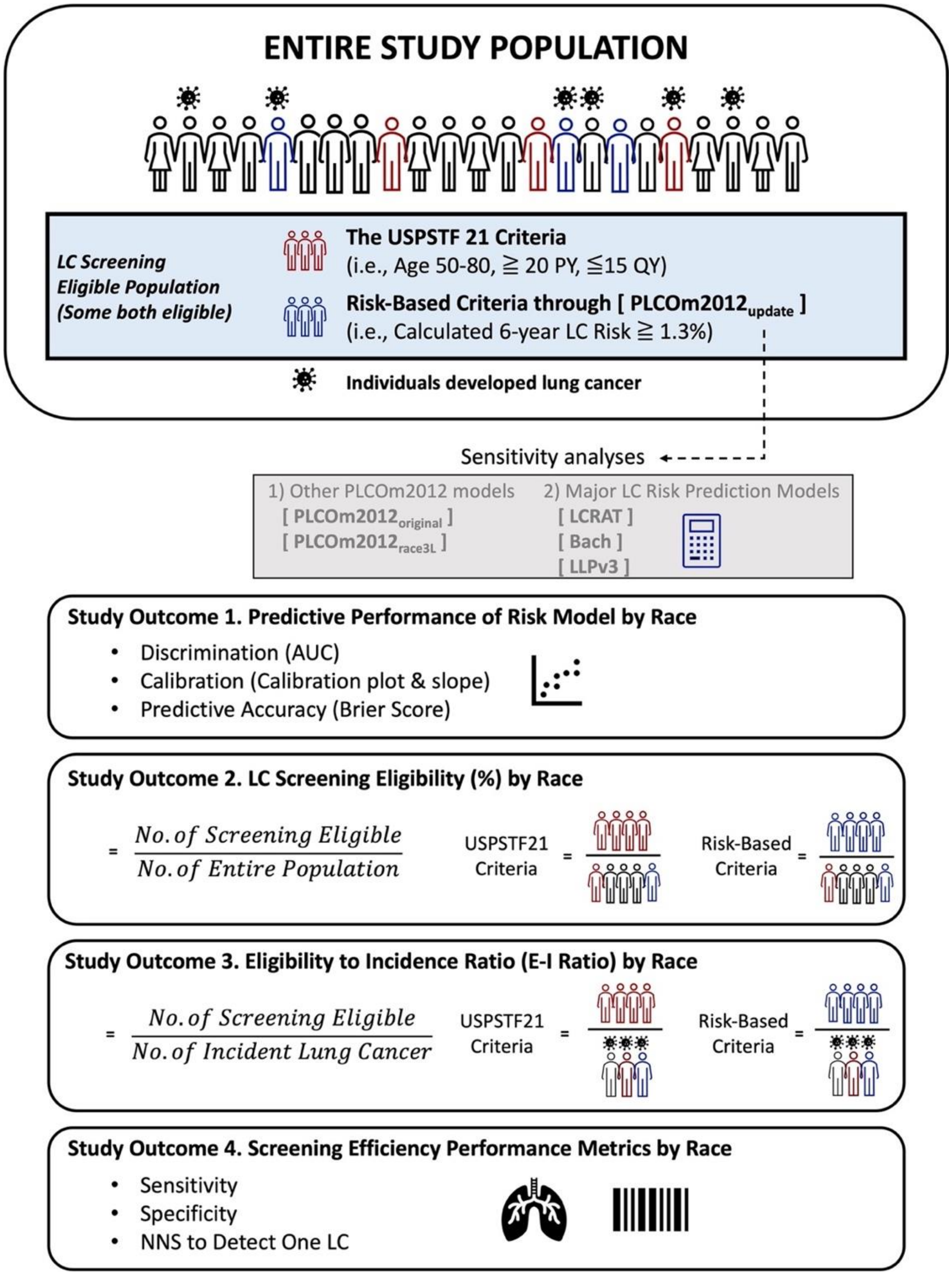

Abbreviations: LC, lung cancer; LCRAT, Lung cancer risk assessment tool; LLPv3, Liverpool Lung Project lung risk stratification tool; NNS, number needed to screen; PLCO, Prostate, Lung, Colorectal, and Ovarian Cancer Screening Trial; USPSTF, the United State Preventive Services Task Force.

© 2023 Choi E et al. *JAMA Oncology*.

eFigure 2. Predictive performance of the PLCOm2012$_{original}$ by race

The discriminatory ability of PLCOm2012$_{original}$ model is evaluated by AUC. Calibration between the observed and predicted probability of developing 6-year lung cancer risk is presented with calibration plot and calibration slope[a]. Smaller Brier score[b] indicates higher predictive accuracy.

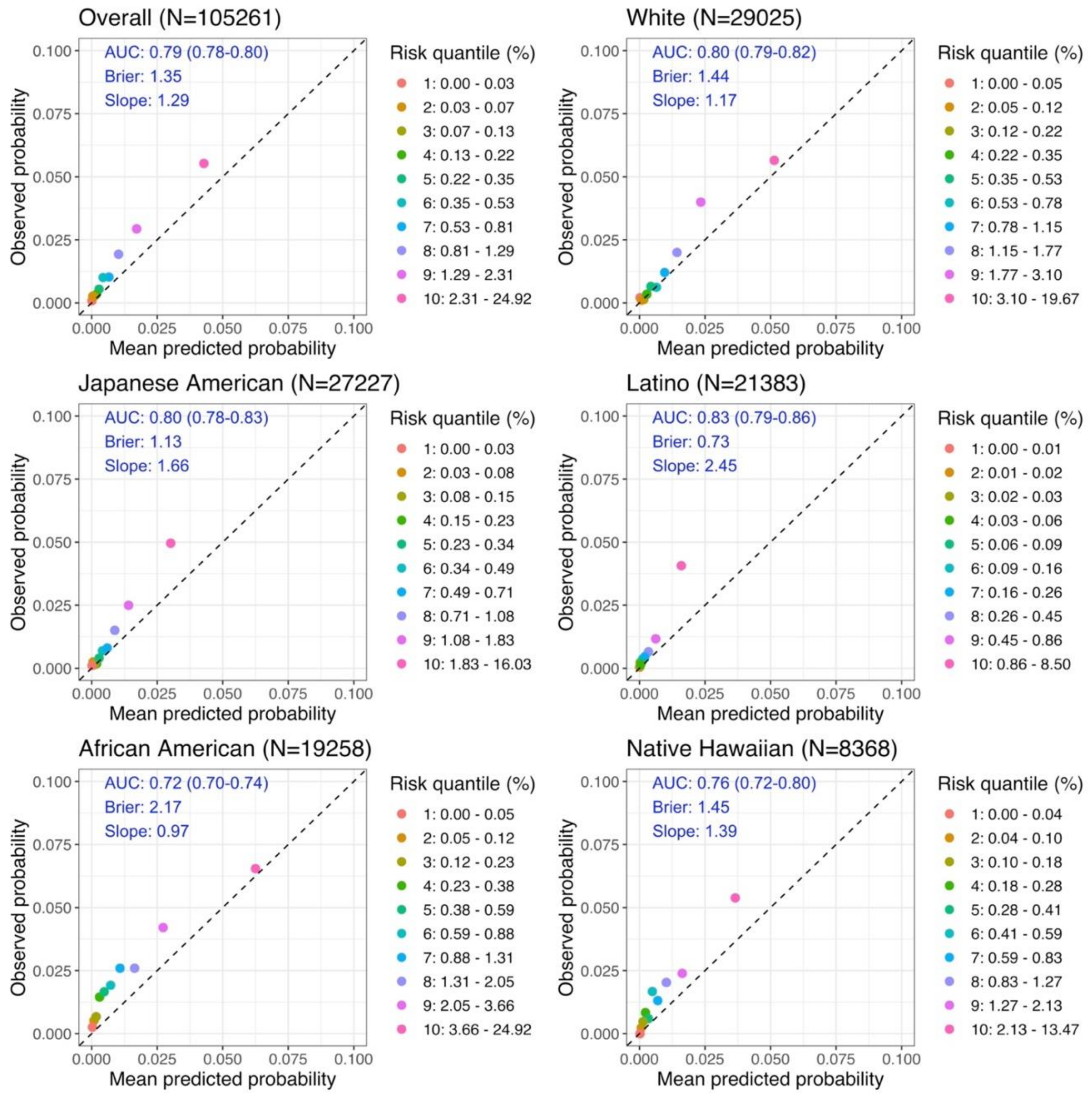

Abbreviation: AUC, area under the receiver operating characteristic curve; PLCO, Prostate, Lung, Colorectal, and Ovarian Cancer Screening Trial.

Notes:

[a] Calibration measures the overall agreement between the observed and predicted outcomes by plotting a calibration curve between the observed versus predicted event status in groups by quantiles (e.g., deciles) of the predicted probabilities. In our study, calibration ability was further quantified using the slope of the fitted linear regression between the means of observed and predicted probabilities across the decile groups. Perfect agreement between observed and predicted probability over deciles is shown by a slope of 1. The calibration slope is a simple, straightforward metric for evaluating overall calibration, but the graphical calibration plot across risk decile groups should also be taken into account because good calibration is dependent on a risk threshold of interest.

[b] Brier score measures the accuracy of probabilistic predictions in the range of 0 and 1. The lower the Brier score, the better the predictions are calibrated.

© 2023 Choi E et al. *JAMA Oncology*.

eFigure 3. Predictive performance of the PLCOm2012$_{update}$ by race

The discriminatory ability of PLCOm2012$_{update}$ is evaluated by AUC. Calibration between the observed and predicted probability of developing a 6-year lung cancer risk is presented with a calibration plot and calibration slope[a]. A smaller Brier score[b] indicates higher predictive accuracy. All estimates are based on 10-fold cross-validation.

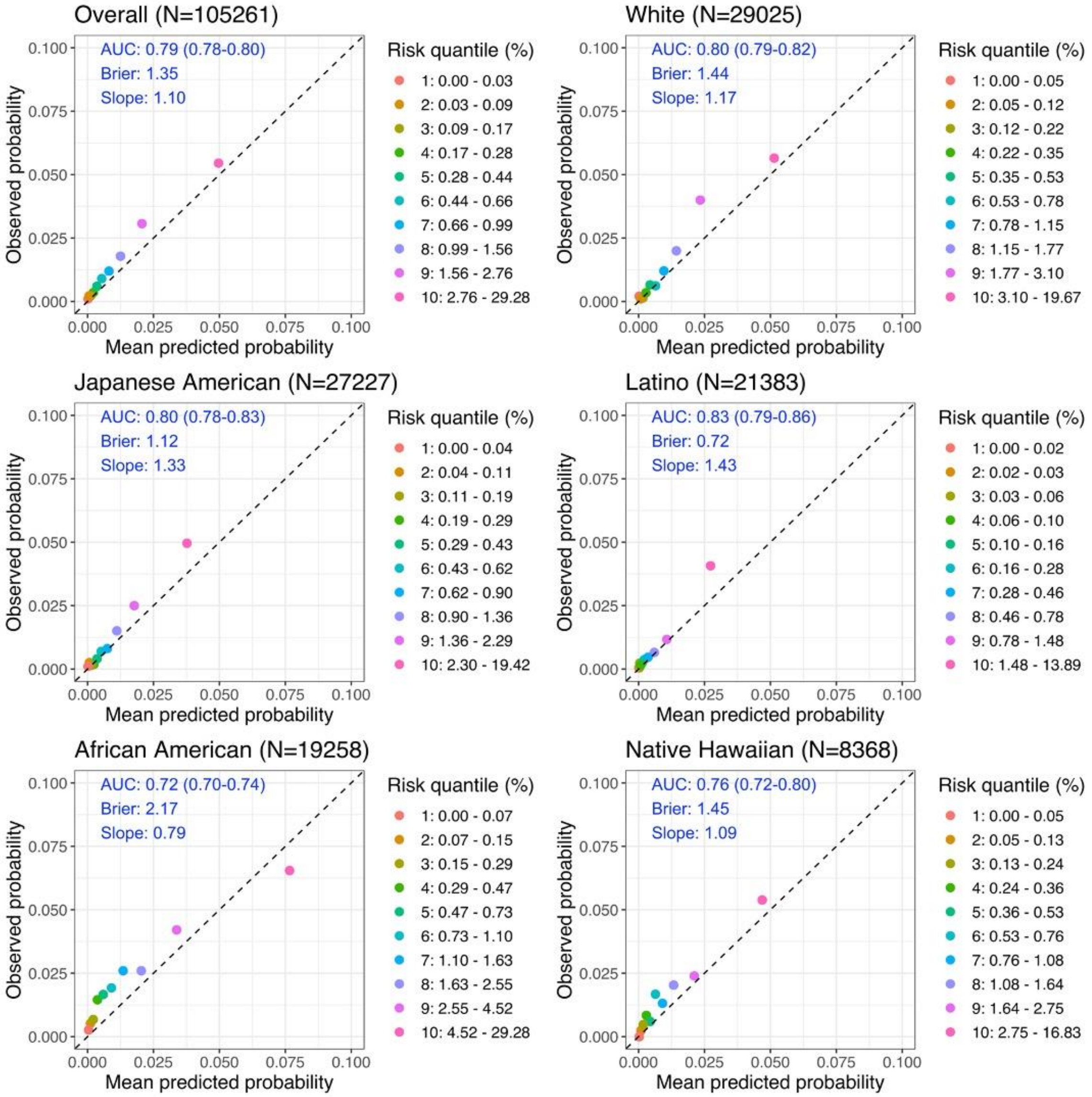

Abbreviations: AUC, area under the receiver operating characteristic curve; PLCO, Prostate, Lung, Colorectal, and Ovarian Cancer Screening Trial.

Notes:

[a] Calibration measures the overall agreement between the observed and predicted outcomes by plotting a calibration curve between the observed versus predicted event status in groups by quantiles (e.g., deciles) of the predicted probabilities. In our study, calibration ability was further quantified using the slope of the fitted linear regression between the means of observed and predicted probabilities across the decile groups. Perfect agreement between observed and predicted probability over deciles is shown by a slope of 1. The calibration slope is a simple, straightforward metric for evaluating overall calibration, but the graphical calibration plot across risk decile groups should also be taken into account because good calibration is dependent on a risk threshold of interest.

[b] Brier score measures the accuracy of probabilistic predictions in the range of 0 and 1. The lower the Brier score, the better the predictions are calibrated

© 2023 Choi E et al. *JAMA Oncology*.

eFigure 4. Predictive performance of the PLCOm2012$_{race3L}$ by race

The discriminatory ability of the model is evaluated by AUC. Calibration between the observed and predicted probability of developing 6-year lung cancer risk is presented with calibration plot and calibration slope[a]. Smaller Brier score[b] indicates higher predictive accuracy.

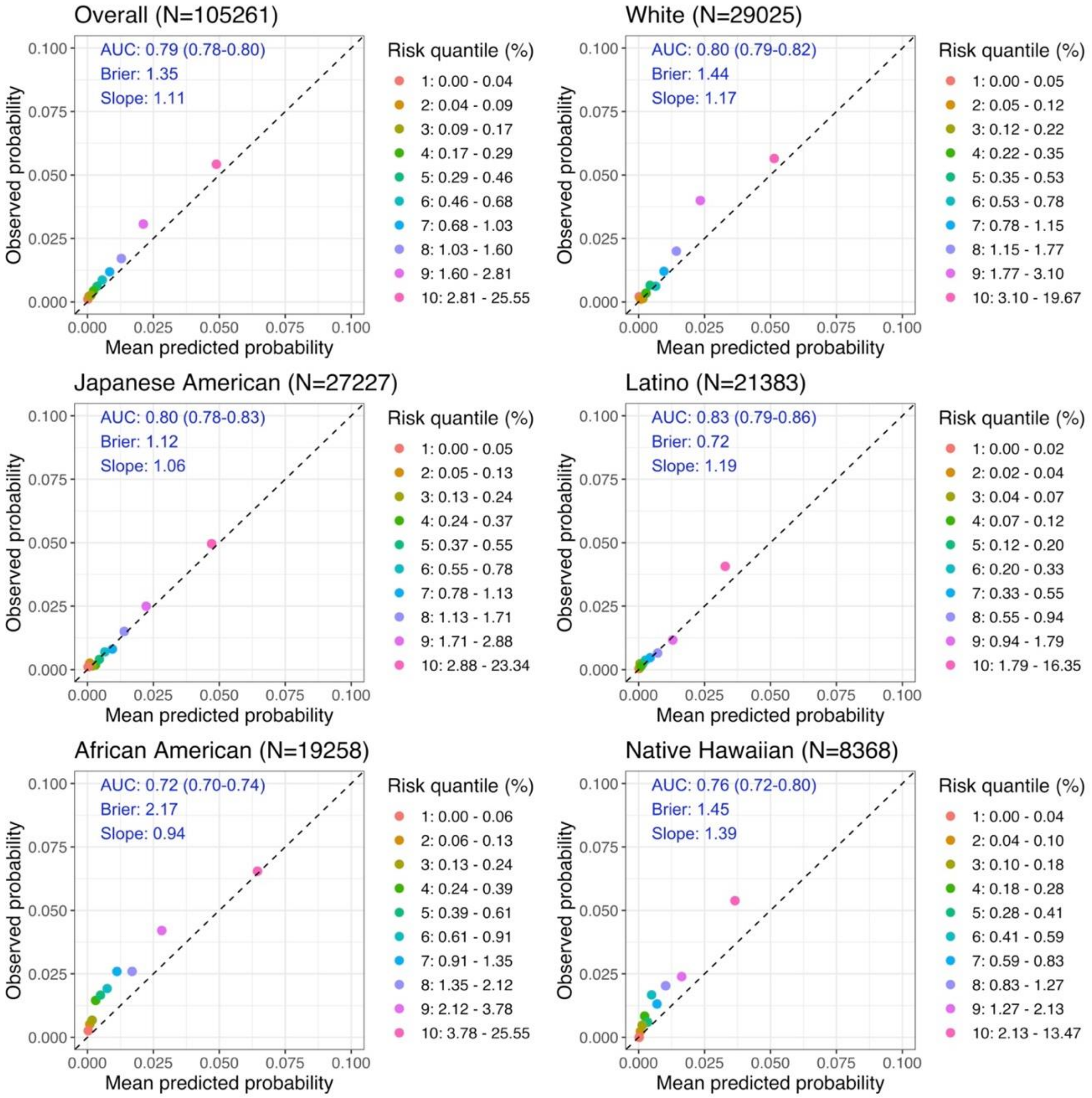

Abbreviation: AUC, area under the receiver operating characteristic curve; PLCO, Prostate, Lung, Colorectal, and Ovarian Cancer Screening Trial.

Notes:

[a] Calibration measures the overall agreement between the observed and predicted outcomes by plotting a calibration curve between the observed versus predicted event status in groups by quantiles (e.g., deciles) of the predicted probabilities. In our study, calibration ability was further quantified using the slope of the fitted linear regression between the means of observed and predicted probabilities across the decile groups. Perfect agreement between observed and predicted probability over deciles is shown by a slope of 1. The calibration slope is a simple, straightforward metric for evaluating overall calibration, but the graphical calibration plot across risk decile groups should also be taken into account because good calibration is dependent on a risk threshold of interest.

[b] Brier score measures the accuracy of probabilistic predictions in the range of 0 and 1. The lower the Brier score, the better the predictions are calibrated

© 2023 Choi E et al. *JAMA Oncology*.

eFigure 5. Predictive performance of LCRAT by race
The discriminatory ability of the model is evaluated by AUC. Calibration between the observed and predicted probability of developing 6-year lung cancer risk is presented with calibration plot and calibration slope[a]. Smaller Brier score[b] indicates higher predictive accuracy.

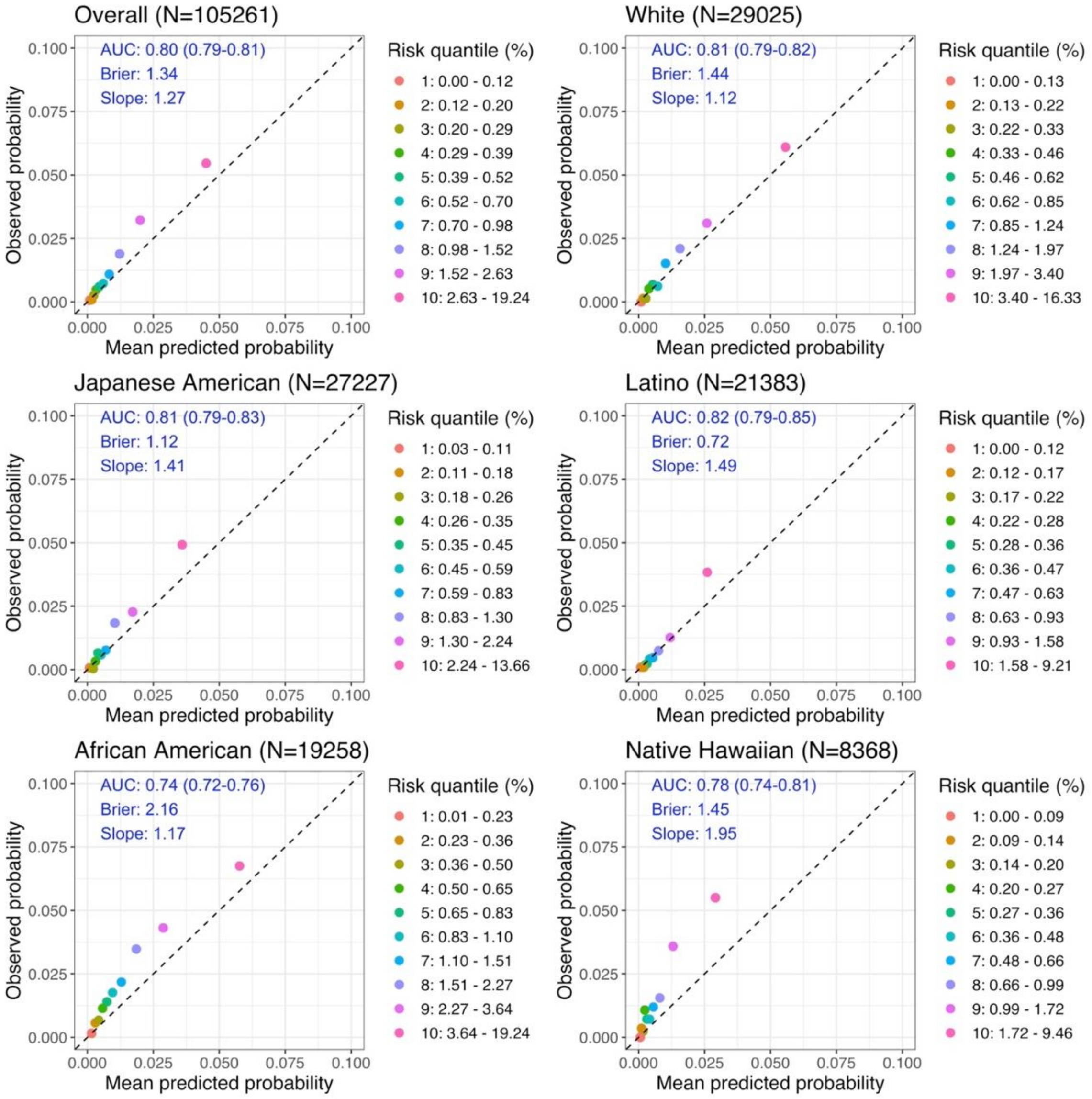

Abbreviation: AUC, area under the receiver operating characteristic curve.
Notes:
[a] Calibration measures the overall agreement between the observed and predicted outcomes by plotting a calibration curve between the observed versus predicted event status in groups by quantiles (e.g., deciles) of the predicted probabilities. In our study, calibration ability was further quantified using the slope of the fitted linear regression between the means of observed and predicted probabilities across the decile groups. Perfect agreement between observed and predicted probability over deciles is shown by a slope of 1. The calibration slope is a simple, straightforward metric for evaluating overall calibration, but the graphical calibration plot across risk decile groups should also be taken into account because good calibration is dependent on a risk threshold of interest.
[b] Brier score measures the accuracy of probabilistic predictions in the range of 0 and 1. The lower the Brier score, the better the predictions are calibrated

© 2023 Choi E et al. *JAMA Oncology*.

eFigure 6. Predictive performance of the Bach model by race
The discriminatory ability of the model is evaluated by AUC. Calibration between the observed and predicted probability of developing 6-year lung cancer risk is presented with calibration plot and calibration slope[a]. Smaller Brier score[b] indicates higher predictive accuracy.

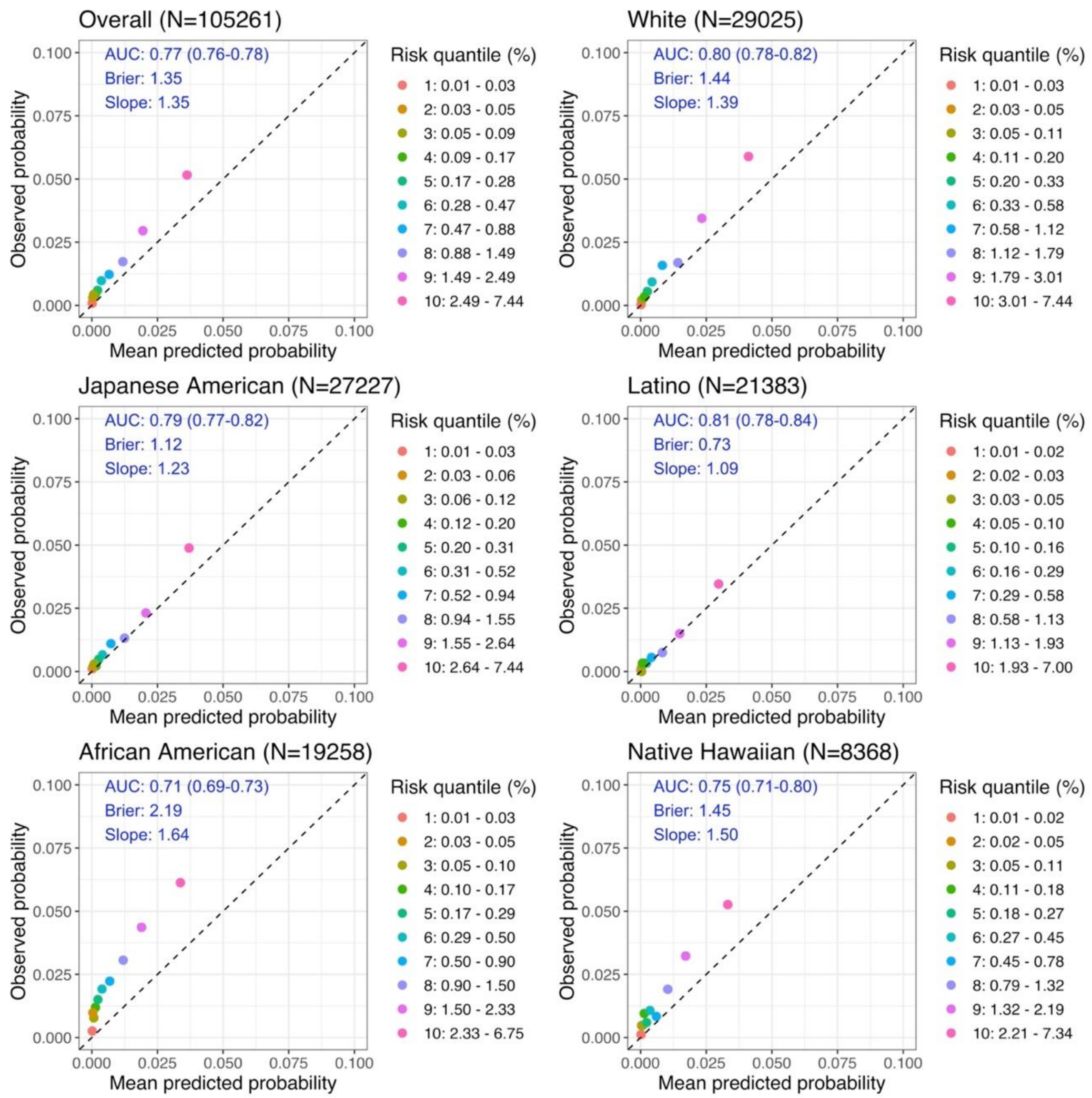

Abbreviation: AUC, area under the receiver operating characteristic curve.
Notes:
[a] Calibration measures the overall agreement between the observed and predicted outcomes by plotting a calibration curve between the observed versus predicted event status in groups by quantiles (e.g., deciles) of the predicted probabilities. In our study, calibration ability was further quantified using the slope of the fitted linear regression between the means of observed and predicted probabilities across the decile groups. Perfect agreement between observed and predicted probability over deciles is shown by a slope of 1. The calibration slope is a simple, straightforward metric for evaluating overall calibration, but the graphical calibration plot across risk decile groups should also be taken into account because good calibration is dependent on a risk threshold of interest.
[b] Brier score measures the accuracy of probabilistic predictions in the range of 0 and 1. The lower the Brier score, the better the predictions are calibrated

© 2023 Choi E et al. *JAMA Oncology*.

eFigure 7. Predictive performance of LLPv3 by race

The discriminatory ability of the model is evaluated by AUC. Calibration between the observed and predicted probability of developing 5-year lung cancer risk is presented with calibration plot and calibration slope[a]. Smaller Brier score[b] indicates higher predictive accuracy.

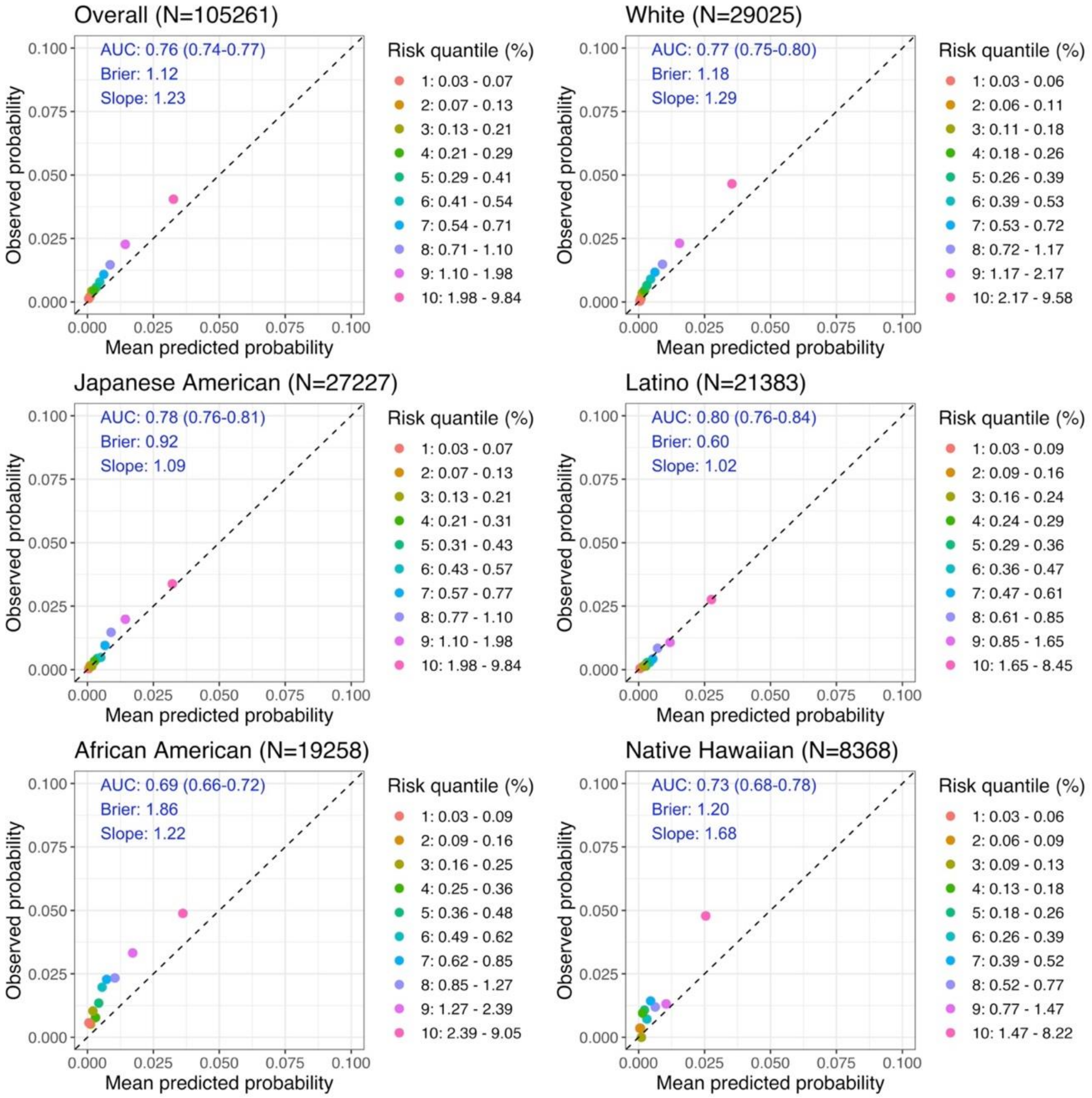

Abbreviation: AUC, area under the receiver operating characteristic curve.

Notes:

[a] Calibration measures the overall agreement between the observed and predicted outcomes by plotting a calibration curve between the observed versus predicted event status in groups by quantiles (e.g., deciles) of the predicted probabilities. In our study, calibration ability was further quantified using the slope of the fitted linear regression between the means of observed and predicted probabilities across the decile groups. Perfect agreement between observed and predicted probability over deciles is shown by a slope of 1. The calibration slope is a simple, straightforward metric for evaluating overall calibration, but the graphical calibration plot across risk decile groups should also be taken into account because good calibration is dependent on a risk threshold of interest.

[b] Brier score measures the accuracy of probabilistic predictions in the range of 0 and 1. The lower the Brier score, the better the predictions are calibrated

© 2023 Choi E et al. *JAMA Oncology*.

eFigure 8. Screening performance (positive and negative predictive value) of the USPSTF 2021 criteria and risk-based screening through the PLCOm2012$_{update}$ (6-year risk≥1.3%) by race

Screening efficiency performance was quantified by positive predictive value (PPV; the number of incident lung cancer cases among screening eligible participants divided by the total number of screening eligible participants) and negative predictive value (NPV; the number of non-lung cancer cases among screening ineligible participants divided by the total number of screening ineligible participants).

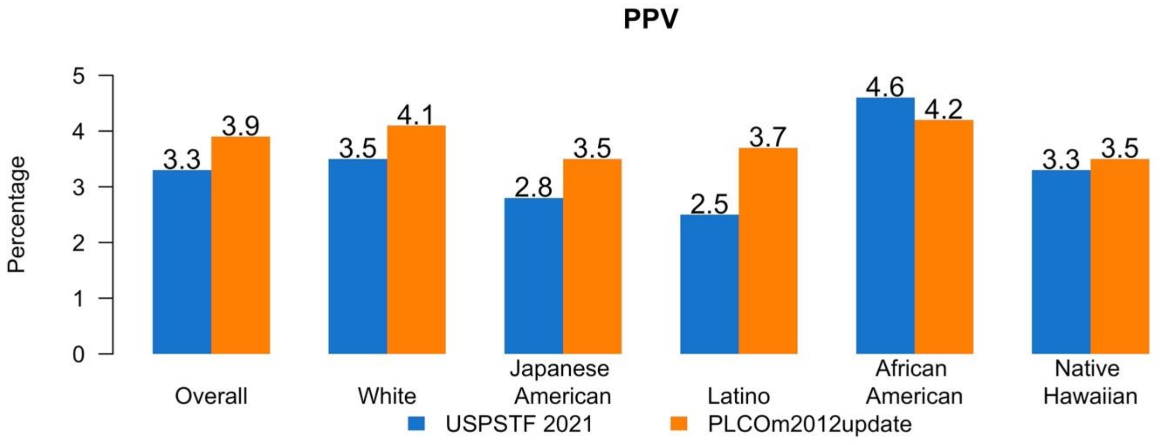

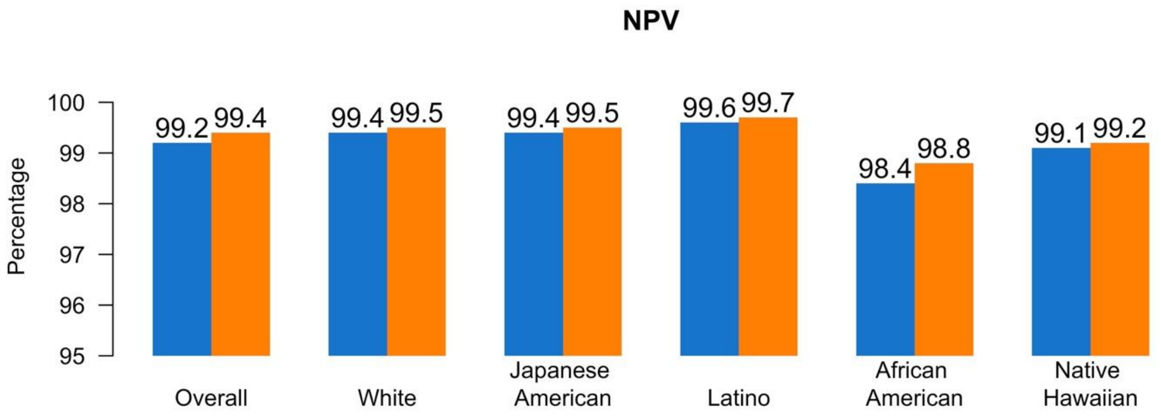

Abbreviation: NPV, negative predictive value; PLCO, Prostate, Lung, Colorectal, and Ovarian Cancer Screening Trial; PPV, positive predictive value; USPSTF, the United States Preventive Services Task Force.

© 2023 Choi E et al. *JAMA Oncology*.

eFigure 9. Sensitivity analysis for evaluating racial disparities in the eligibility to Incidence (E-I) ratio through the USPSTF 2021 and risk-based screening (PLCOm2012$_{original}$ [6-year risk≥1.1%] and PLCOm2012$_{race3L}$ [6-year risk≥1.3%])
Screening eligibility to 6-year lung cancer incidence (E-I) ratio is compared between the USPSTF 2021 criteria and risk-based criteria (PLCOm2012$_{original}$ and PLCOm2012$_{race3L}$) across different races and ethnicities. The risk thresholds of the PLCOm2012original (≥1.1%) and PLCOm2012race3L (≥1.4%) were chosen to match the eligibility level of the USPSTF 2021 criteria to the MEC data. The percent different of E-I ratio between White and other racial groups are calculated as follows: *[(E-I ratio of White – E-I ratio of another racial group)/(E-I ratio of White)x100]*.

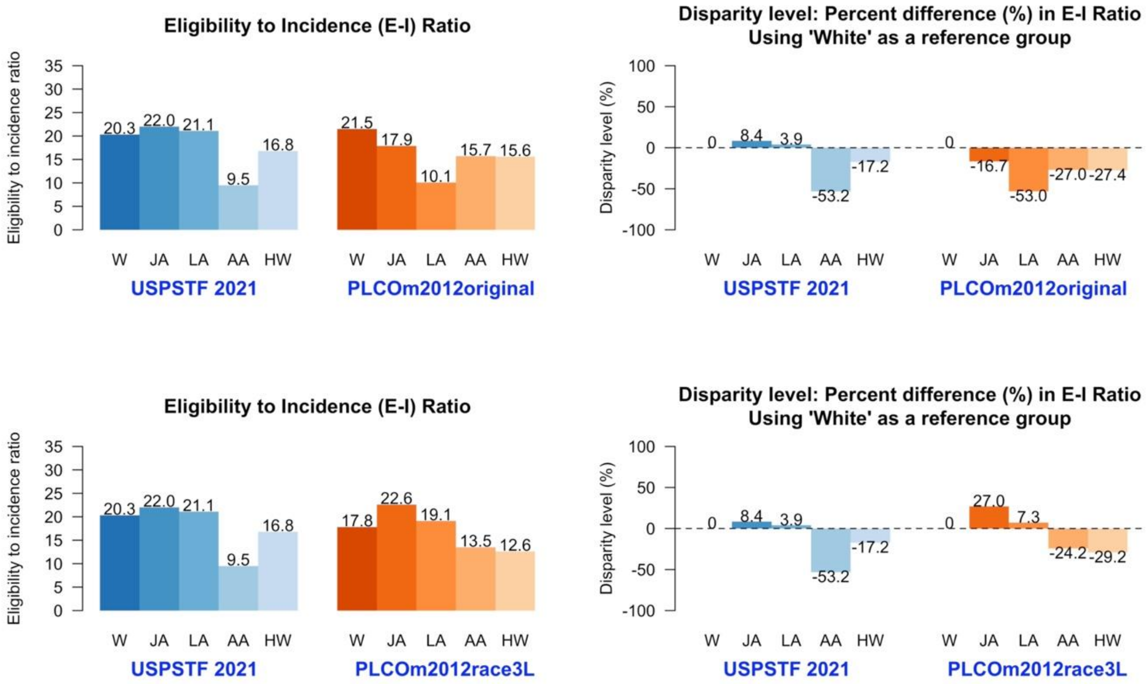

Abbreviations: AA, African American; HW, Native Hawaiian; JA, Japanese American; LA, Latino; PLCO, Prostate, Lung, Colorectal, and Ovarian Cancer Screening Trial; USPSTF, the United States Preventive Services Task Force; W, White.

© 2023 Choi E et al. *JAMA Oncology*.

eFigure 10. Sensitivity analysis for screening performance through the USPSTF 2021 and risk-based screening (PLCOm2012$_{original}$ [6-year risk≥1.1%] (A) and PLCOm2012$_{race3L}$ [6-year risk≥1.4%]) (B)

The risk thresholds of the PLCOm2012original (≥1.1%) and PLCOm2012race3L (≥1.4%) were chosen to match the eligibility level of the USPSTF 2021 criteria to the MEC data.

(A) The USPSTF 2021 vs. risk-based screening through PLCOm2012$_{original}$ (6-year risk≥1.1%)

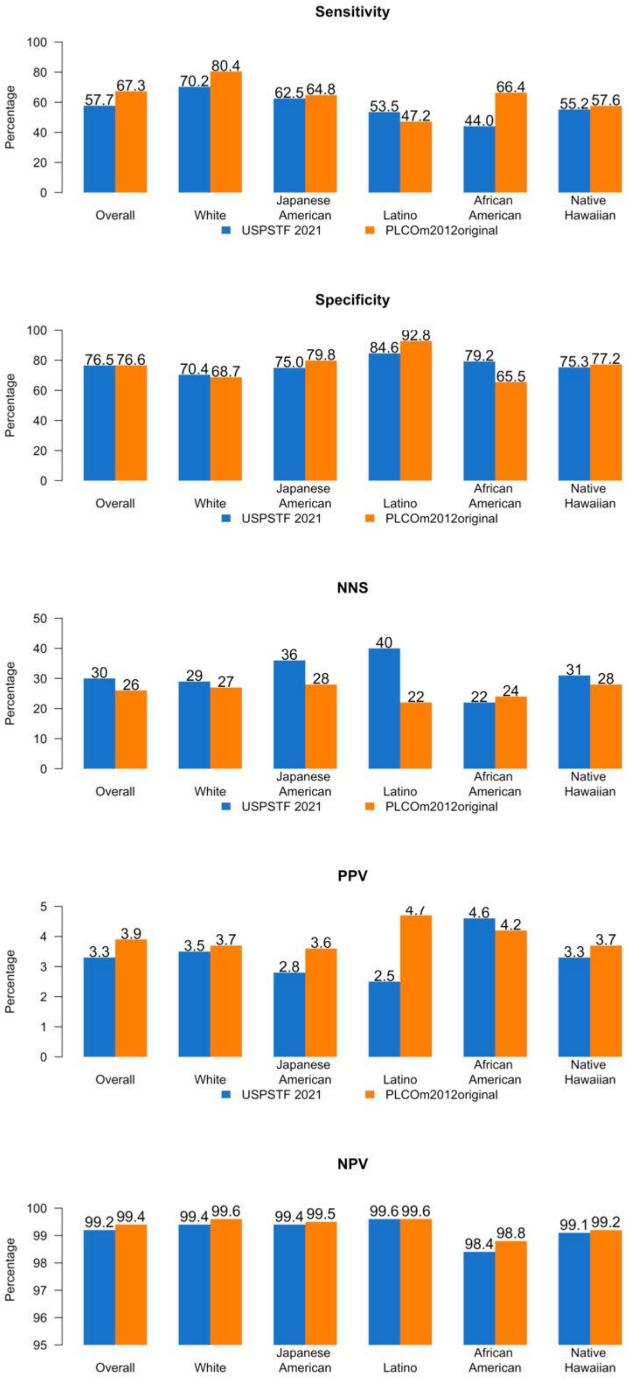

© 2023 Choi E et al. *JAMA Oncology*.

(B) The USPSTF 2021 vs. risk-based screening through $PLCOm2012_{race3L}$ (6-year risk≥1.3%)

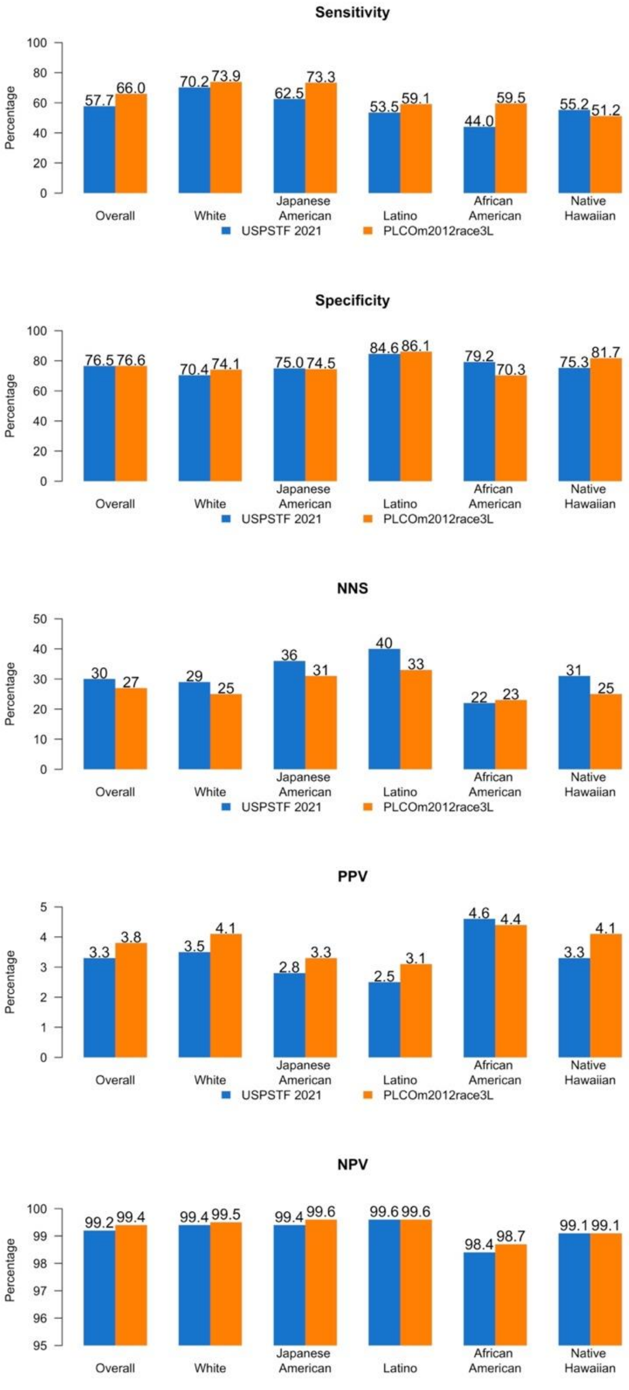

Abbreviations: PLCO, Prostate, Lung, Colorectal, and Ovarian Cancer Screening Trial; USPSTF, the United States Preventive Services Task Force.

© 2023 Choi E et al. *JAMA Oncology*.
