# Supplementary material for: Risk Model–Based Lung Cancer Screening and Racial and Ethnic Disparities in the US
Source: JAMA Oncol. 2023 Oct 26;9(12):1640–8. doi: 10.1001/jamaoncol.2023.4447 (PMC10603577; doi:10.1001/jamaoncol.2023.4447)
Supplement: Supplement 2. — Data Sharing Statement [file jamaoncol-e234447-s002.pdf]

Data Sharing Statement

Choi. Risk Model–Based Lung Cancer Screening and Racial and Ethnic Disparities in the US. *JAMA Oncol*. Published October 26, 2023. doi:10.1001/jamaoncol.2023.4447

Data
Data available: No

Additional Information
Explanation for why data not available: The data underlying this analysis were provided by the Multiethnic Cohort Study (MEC) under data use agreement. Researchers interested in the MEC data may submit an inquiry online: https://www.uhcancercenter.org/for-researchers/mecdata-sharing.
